# Do Contaminants Originating from State-of-the-Art Treated Wastewater Impact the Ecological Quality of Surface Waters?

**DOI:** 10.1371/journal.pone.0060616

**Published:** 2013-04-08

**Authors:** Daniel Stalter, Axel Magdeburg, Kristin Quednow, Alexandra Botzat, Jörg Oehlmann

**Affiliations:** 1 Department Aquatic Ecotoxicology, Biological Sciences Division, Goethe University Frankfurt am Main, Frankfurt am Main, Germany; 2 Analytical Environmental Chemistry, Institute for Atmospheric and Environmental Sciences, Goethe University Frankfurt am Main, Frankfurt am Main, Germany; 3 Department of Ecology – Conservation Ecology, Faculty of Biology, Philipps University of Marburg, Marburg, Germany; Federal University of Rio de Janeiro, Brazil

## Abstract

Since the 1980s, advances in wastewater treatment technology have led to considerably improved surface water quality in the urban areas of many high income countries. However, trace concentrations of organic wastewater-associated contaminants may still pose a key environmental hazard impairing the ecological quality of surface waters. To identify key impact factors, we analyzed the effects of a wide range of anthropogenic and environmental variables on the aquatic macroinvertebrate community. We assessed ecological water quality at 26 sampling sites in four urban German lowland river systems with a 0–100% load of state-of-the-art biological activated sludge treated wastewater. The chemical analysis suite comprised 12 organic contaminants (five phosphor organic flame retardants, two musk fragrances, bisphenol A, nonylphenol, octylphenol, diethyltoluamide, terbutryn), 16 polycyclic aromatic hydrocarbons, and 12 heavy metals. Non-metric multidimensional scaling identified organic contaminants that are mainly wastewater-associated (*i.e.*, phosphor organic flame retardants, musk fragrances, and diethyltoluamide) as a major impact variable on macroinvertebrate species composition. The structural degradation of streams was also identified as a significant factor. Multiple linear regression models revealed a significant impact of organic contaminants on invertebrate populations, in particular on Ephemeroptera, Plecoptera, and Trichoptera species. Spearman rank correlation analyses confirmed wastewater-associated organic contaminants as the most significant variable negatively impacting the biodiversity of sensitive macroinvertebrate species. In addition to increased aquatic pollution with organic contaminants, a greater wastewater fraction was accompanied by a slight decrease in oxygen concentration and an increase in salinity. This study highlights the importance of reducing the wastewater-associated impact on surface waters. For aquatic ecosystems in urban areas this would lead to: (i) improvement of the ecological integrity, (ii) reduction of biodiversity loss, and (iii) faster achievement of objectives of legislative requirements, *e.g.*, the European Water Framework Directive.

## Introduction

In urban areas, the water quality of small streams is predominantly impacted by structural degradation of stream morphology (*e.g.*, channelization and straightening), agricultural land use in the catchment, and a high load of treated wastewater (WW). These are also the most apparent drivers of ecological conditions in anthropogenically disturbed surface waters. Together, these stressors have led to significant declines in aquatic fauna populations and biodiversity, in particular in freshwater ecosystems, which in turn has had a profound impact on the ecological integrity of many aquatic ecosystems [1]–[5].

The ecological quality of surface water is commonly assessed by analyzing benthic macroinvertebrate species assemblages [6]. The highly diverse group of aquatic invertebrate organisms encompasses many euryoecious and stenoecious species [7]. The latter have narrow ecological requirements in terms of physico-chemical water parameters and structural characteristics of the water body. Consequently, assessing the invertebrate community can provide a picture of the ‘health’ of the water body and can be used to identify factors affecting its ecological quality (*e.g.*, structural deterioration, acidification, oxygen deficiency). The technique involves comparing the composition and abundance of the present invertebrate taxa to established reference communities from anthropogenically least-disturbed reference sites.

Evaluating and improving the ecological status of surface waters is increasingly perceived to be important, which is reflected by legislation changes in this area [6], [8]. For this purpose, worldwide many different macroinvertebrate-based rating systems for ecological status evaluation with hundreds of indices, metrics, and evaluation tools have been developed [6], [8]. In the European Union, the Water Framework Directive (WFD) [9]–enacted in 2000–set an objective that all coastal and inland waters of Europe be of a ‘good status’ by 2015. One tool for evaluating the quality of these waters is the ASTERICS software (AQEM/STAR Ecological River Classification System), which assists the assessment of ecological status. If the outcomes of ASTERICS analysis indicate a poor ecological status, key causal factors and mitigation and remediation options must be identified and explored in order to achieve the ‘good status’ benchmark.

Until the early 1980s in Germany, the primary driver of the deterioration of aquatic ecosystems was the large input of nutrients via WW discharge [10]. However, as progressive WW treatment and other restoration measures increased throughout the 1980s, nutrient inputs were diminished, eutrophication reduced, and water quality impacts were ameliorated, hence allowing aquatic ecosystems to recover [11]. Generally, in high-income countries, eutrophication is largely under control at present [12], [13] because of the advances in, and the prevalence of WW treatment [14].

Despite the effective removal of nutrients and other contaminants, treated effluent often contains trace organic contaminants that have been demonstrated to have negative impacts on aquatic ecosystems [13]. These anthropogenic water pollutants and their transformation products are often present at low to very low concentrations (*e.g.*, ng/L). Despite such minimal trace level concentrations, the large spectrum of organic contaminants occurring in surface waters [15] may pose a potential threat to aquatic wildlife, particularly with regard to mixture toxicity. Pharmaceuticals and personal care products in particular often exhibit high biological activity–some of which are also persistent in the environment [16], thus giving them the potential to affect aquatic species [17]–[23]. The presence of these contaminants in urban surface water has been primarily attributed to environmental releases of municipal and industrial WW [13]. Accordingly, we anticipate there to be a clear negative impact on macroinvertebrate assemblages and the general ecological status of surface waters in proximity to WW treatment plants.

Many studies have addressed the impact of water pollution on invertebrate communities, but frequently they consider only severely contaminated sites or individual pollutants (*e.g.*, [24]). In this study, we have instead focused on aquatic invertebrate exposure to the ‘subtle pollution’ (*i.e.,* mixtures of organic micropollutants) occurring in German lowland streams. All treatment plants currently operating in the study area are equipped with biological activated sludge treatment–the most widely used WW treatment system in high-income countries [25]. As well as the elimination of nutrients, this technology has also been shown to effectively reduce the contaminant load and toxicity of WW [26], [27]. However, a considerable fraction of toxic contaminants remains prevalent in WW treated by biological activated sludge [27].

This study aimed to enhance the understanding of factors that disturb the ecological integrity of surface waters in order to assist decision-making around remediation and mitigation of factors affecting the quality of surface waters. The primary objective was to evaluate whether lowland stream macroinvertebrate assemblages are affected by their loads of WW-associated trace organic contaminants (OC) in the water phase. Additionally, the potential impacts of heavy metals (HM) and polycyclic aromatic hydrocarbons (PAH) in sediment were investigated.

## Materials and Methods

### Study Area

The study was conducted in the Hessian Ried (HR) close to Frankfurt (Main), Germany. The region extends about 1200 km^2^ and is bound by the river Main in the South, the lower mountain range Odenwald in the East, and the river Rhine in the West. The accumulation of sand and gravel, as a result of aggradational deposits, forms a large aquifer that serves as drinking water reservoir for the Frankfurt/Rhine-Main conurbation [28]. The population of the HR is approximately 800,000. Types of land use include intensive agriculture (predominantly cultivation of vegetables, fruit, wheat, and forage crops) and industry (*e.g.*, chemical and manufacturing industries), and high-density traffic highways occur in the region [28]. Due to this high level of anthropogenic disturbance, the importance of the aquifer in the region, and the homogenous distribution of urbanization, the HR was selected as a representative urban study area to investigate the impacts of water pollution, physico-chemical factors, and structural characteristics on the ecological quality of urbanized aquatic ecosystems.

### Study Design

To identify key impact factors on the ecological quality, we assessed the benthic macroinvertebrate community of the streams, recorded a range of environmental and anthropogenic variables, and evaluated their potential impact on the species composition with the major focus on wastewater-associated contaminants. We conducted this study from 2005 to 2006 at 26 sampling sites in four river systems that run through the study area in an east-west direction and discharge into the Rhine River (Table 1). Most sampling sites belong to the water body type 19 (small streams in floodplains) according to the WFD [9], [29]. Exceptions are sampling sites 1 to 6 in the Weschnitz stream (type 9: mid-sized siliceous rivers in the lower mountain ranges with fine to crude sediment) as well as Sw1 (type 6: small calcareous sediment rich streams in the lower mountain ranges) and Mo1 (type 5: small siliceous cobble-bottom streams in the lower mountain ranges [30]). 28 sewage treatment plants are currently operating in the catchment. The location of the sampling sites relative to these plants, as well as the quantity of effluent discharged to streams, resulted in different WW loads at each of the sampling sites. This factor was defined on a scale from 0–100% (Figure S1–4). The only sampling site without WW was Wi1.

**Table 1:** River systems with respective streams, abbreviations (abbr.), and number of sampling sites (no.).

river systems	streams	abbr.	no.
Schwarzbach-Landgraben-system	Schwarzbach, Apfelbach	Sw	6
Schwarzbach-Landgraben-system	Landgraben, Darmbach	La	2
Modau-Sandbach-system	Sandbach	Sa	3
Modau-Sandbach-system	Modau	Mo	4
Winkelbach	Winkelbach	Wi	5
Weschnitz	Weschnitz	We	6

### Structural Quality of the Streams

To classify the streams with respect to their structural modification, we recorded the morphology or water structure quality following Zumbroich *et al.* [31] from class 1 (near-natural) to class 7 (completely modified). Additionally, the stream bed structure was characterized in detail (relative coverage of 10 different sediment types, ) according to the macroinvertebrate sampling procedure described in Hering *et al.* [7].

### Benthic Macroinvertebrate Community

We sampled the invertebrates in autumn and spring (September 2005 and March 2006) following the multi-habitat sampling procedure described in Hering *et al.* [7]. No specific permits were required for the described field studies as none of the sampling sites are privately-owned or protected in any way. The responsible Hessian State Office for Environment and Geology was informed prior to the study. Before sampling, we assessed the substrate composition of the sampling sites of a 100-m transect of each water body. The different substrate types were sampled according to their relative coverage with a total of 20 sub-samples per sampling site. We used a Surber-sampler (manufactured by precision engineers at the Goethe University of Frankfurt, Germany) with 500-µm mesh size and 20×25-cm frame size for macroinvertebrate sampling, resulting in 1-m^2^ sampling plots per sampling site. Endangered or protected species were identified in the field and immediately released back to the streams. The remaining sample material was stored in 70% ethanol (denatured, Carl Roth GmbH, Darmstadt, Germany). Macroinvertebrates were separated as described in Haase & Sundermann [32] and determined to species level, if possible, but at least to the minimum taxonomic level recommended by Haase *et al.* [33] (Table S2–3). For separation and identification of the species we used stereo microscopes (Stemi 2000, Carl Zeiss AG, Oberkochen, Germany) and a microscope (BX 50, Olympus, Tokio, Japan) if a higher magnification was required, *e.g.*, for the identification of Ephemeroptera species. We could not sample Mo4 and Sw6 during the autumn sampling campaign due to high water levels.

### Contaminants

As WW-associated pollutants commonly correlate in their occurrence, distinguishing their individual impacts is hardly feasible. Accordingly, a number of representative pollutants were selected to account for the burden of WW-associated OCs in general. At 11 sampling campaigns, water samples from each sampling site were analyzed for 12 common organic pollutants: five organophosphates; two musk fragrances; bisphenol A; the alkylphenols, nonylphenol and octylphenol; and the insect repellent diethyltoluamide (Table 2). Pollutants were selected due to their ubiquitous occurrence in municipal WW. Unexpectedly, terbutryn–in Europe formerly used as agricultural herbicide, which is still authorized as a biocide in antifouling paints and coatings–was detected in all water samples from the beginning of the sampling campaigns, and therefore was also included in the analysis suite. For more detailed information on analytical procedures and pollutant concentrations, see Quednow & Puettmann [34]–[37]. To take into account a possible influence of sediment-bound pollutants, the sediment load of 12 HMs and metalloids as well as 16 PAHs (Table 2)–the latter selected as proposed by the US EPA [38]–was determined according to standard methods [39], [40]. Average contaminant concentrations are given in Table S4–6.

**Table 2:** Analyzed contaminants.

phase analyzed	contaminant group	contaminants
**water**	organophosphates	TBP (tributyl phosphate), TBEP (tris(2-butoxyethyl)phosphate), TCEP (tris(2-chloroethyl)phosphate),TCPP (tris(2-chloro-, 1-methyl-ethyl)-phosphate), TDCPP (tris(1,3-dichloro-2-propyl) phosphate)
	biphenols	BPA (bisphenol A)
	musk fragrances	HHCB (1,3,4,6,7,8-hexahydro-4,6,6,7,8,8-hexamethylcyclopenta-γ-2-benzopyran), AHTN (6-Acetyl-1,1,2,4,4,7- hexamethyltetraline)
	alkylphenols	octylphenol, nonylphenol
	triazines	terbutryn
	amides	DEET (diethyltoluamide)
**sediment**	heavy metals	aluminium, arsenic, barium, cadmium, cobalt, chromium, copper, iron, manganese, nickel, lead, zinc
	polycyclic aromatic hydrocarbons	naphthalene, acenaphtylene, acenaphthene, fluorene, phenanthrene, anthracene, fluoranthene, pyrene, benzo(a)anthracene, chrysene, benzo(b)fluoranthene, benzo(k)fluoranthene, benzo(a)pyrene, dibenz(ah)anthracene, benzo(ghi)perylene, indeno(1,2,3-cd)pyrene

### Water and Sediment Quality

We determined the organic carbon content and average grain size of sediments according to standard methods [41], [42]. We recorded conductivity, temperature, O_2_-concentration and pH (using multiparameter instrument Multi 350i, WTW, Weilheim, Germany) as well as flow velocity (using a hydrometric vane from OTT Hydromet, Kempten, Germany) during sampling events. Colorimetric on-site tests (Aquaquant®, Merckoquant®, Merck, Darmstadt, Germany) were used to determine ammonium, chloride, phosphate, total hardness, and carbonate hardness. Biological oxygen demand (BOD_5_) was analyzed according to a standard method [43].

### Data Analysis

We used the ASTERICS software [44] to calculate the ecological status (from 1–5: bad, poor, moderate, good, high) and biotic metrics (*e.g.*, saprobic index, Shannon index, Simpson index, number of EPT taxa) for each sampling site. To reduce the number of contaminant variables, separate principal component analyses (PCAs) were performed using SPSS (version 14, SPSS Inc., Chicago, USA, 2005) with the concentrations of OCs in the water phase and concentrations of PAHs and HMs in the sediments (Table S7–9). We only considered components with an eigenvalue >1 to limit the number of resulting components. The component scores were determined by regression and were standardized with a mean of 0 and standard deviation of 1 [45]. The resulting components (OC1–4, HM1–3, and PAH1–2) with the corresponding component scores were used to replace the contaminant concentrations in all further analyses.

Using the statistic software R (R Development Core Team 2011) we applied non-metric multidimensional scaling (NMDS) using Bray-Curtis dissimilarity as implemented in R package vegan 2.0–2 [46] based on abundance data to detect differences of macroinvertebrate taxa composition among the 26 sample sites. NMDS displays dissimilarities in community composition nonlinearly onto ordination space, can cope with nonlinear species responses, and is not constrained by predictors [46]. We excluded rare species that occurred less than three times and that were present in less than three sampling sites. We used taxa scores in the biplot to illustrate differences between sampling sites. Non-correlated variables were selected (structural quality, average discharge, organic carbon content of the sediments, and following contaminant factors: OC1, OC2, HM1, HM3, PAH1, PAH2; Table S1) and were fitted post-hoc to the ordination and their significance was tested via random permutations (1000 iterations).

We used multiple linear regression models to test the impact of WW-associated contaminants and environmental variables on the following dependent variables: total abundance of macroinvertebrates, number of macroinvertebrate taxa, Simpson and Shannon diversity of macroinvertebrates, number of EPT taxa, and saprobic index. We started with models comprising the same set of non-correlated variables as used for the NMDS. We used the step function implemented in R for model selection to reach minimum adequate models. This selection process is based on minimizing information loss according to Akaike’s Information Criterion values [47].

We performed Spearman rank correlation analyses using species abundances, biotic metrics, the PCA-derived contaminant factors, and all environmental variables (Table S1). To take into account the toxicity of the analyzed WW-associated contaminants, toxic units (TUs) were calculated as described in Liess & Von Der Ohe [48] and included in the correlation analysis. The TU values are based on effect concentrations (LC_50_ and ‘no observed effect concentration’) from acute tests with *Chirmonomus riparius* and chronic tests with *Daphnia magna*). In addition to the median pollutant concentrations, the 90^th^ percentile of the concentrations was used for TU calculations to estimate whether the peak loads influenced the taxa composition more than the mean concentrations. TUs were summed for each sampling site and included in the correlation analysis.

## Results

### Sampling Sites and Species Data

The running waters of the study area were mostly structurally distorted with channel-like character, usually with trapezoidal profiles, low depth variance, large profile depth, and almost a lack of curvature erosion. Accordingly, the degree of anthropogenic deformation varied mostly between class 4 (significantly modified) to 7 (completely modified) with only La2 and Mo1 as slightly modified (class 2) and Sw1 as single near-natural stream (class 1; Figure S5). We identified a total of 141 different taxa across all sampling sites (Table S2–3). The number of taxa exceeded 50 at Wi1 and Wi2 only, while falling below 20 at La1 and Sw6 (Figure S5). The lowest Shannon diversity was determined for La1 (0.8) and the highest for Sw1 (3.0). At La1 and Sw2, the invertebrate abundance was dominated by Oligochaeta and *Gammarus roeselii,* contributing more than 50% to the total number of individuals (dominance structure in Figure S5 given as contribution of individual taxa to the total number of individuals per m^2^ in decreasing order). Plecoptera were found at Sw1 and We1 only. Invasive species like *Corbicula fluminea*, *Potamopyrgus antipodarum*, and *Dikerogammarus villosus* were commonly found in the sampling area. At Sa2 *P. antipodarum* dominated species assemblage in terms of individuals per m^2^. The ecological status was evaluated as ‘good’ for two sampling sites only (Wi2 and Wi4), while 50% of the sites were evaluated as insufficient or poor (Figure S5).

### PCA-derived Variable Reduction of Contaminants

Four components of the PCA of OCs explained *ca.* 88% of the total variance while component loadings (*i.e.,* correlation coefficients between the variables and components) ranged from 0.55–0.97. The first component (OC1) encompassed the organophosphates as well as the musk fragrances and DEET (Table S7). OC1 is the contaminant factor that correlated to the highest degree with the WW load (Spearman’s ρ = 0.932, Table S1) and is therefore regarded as predominantly WW-associated. Octylphenol and terbutryn were mainly represented by the second component (OC2), bisphenol A by the third (OC3), and nonylphenol by the fourth (OC4) component. The three components of HMs explained *ca.* 78% of the total variance. The component loadings ranged from 0.64–0.92. The first component (HM1) represented primarily Co, Cr, Cu, Fe, Mn, Ni, Pb, and Zn (Table S8) while the second (HM2) largely encompassed Al, As, and Ba. The third component (HM3) represented almost exclusively Cd. The two components calculated for PAHs explained more than 92% of the total variance, whereas component loadings were mainly >0.9 (except for Acyl and Fl, Table S9). The first component (PAH1) embodied 14 of the 16 PAHs while the second (PAH2) represented acenaphtylene and fluorine. PCA reduced the total number of contaminant variables from 40 variables to 9 variables, which were then used for subsequent analyses.

### Dissimilarities in Macroinvertebrate Assemblages Related to Structural Degradation and Contaminants

**Figure 1:**
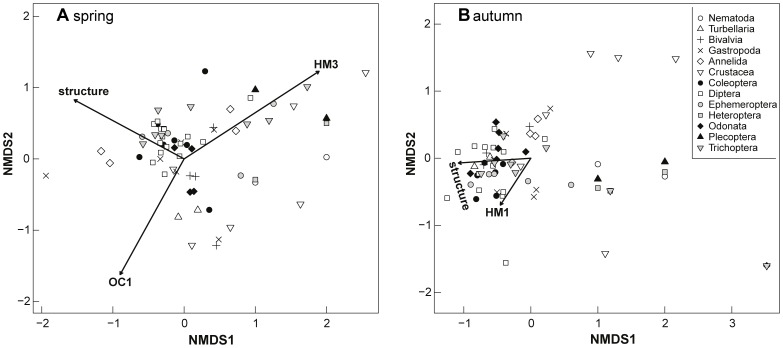
NMDS biplot of taxa and environmental variables. Displayed are variables with a significant impact (p<0.05) for sampling campaign in spring (A) and autumn (B). HM, components of the principal component analysis (PCA) with heavy metals; OC, components of the PCA with organic contaminants; structure, structural degradation. Spring: two convergent solutions, two dimensions, stress = 0.17; autumn: two convergent solutions, two dimensions, stress = 0.21).

The NMDS revealed the water body structure as one of the major impact variables in both sampling campaigns (R^2^ = 0.65 and 0.56; p = 0.005 and 0.002; Figure 1A, B). Other significant impact factors for the taxa composition in spring included OC1 (R^2^ = 0.43, p = 0.006) and HM3 (R^2^ = 0.72, p = 0.005). In the autumn sampling event, HM1 was identified as a significant impact factor (R^2^ = 0.33, p = 0.014), while OC1 and HM3 showed no significant effect (OC1: R^2^ = 0.18, p = 0.11; HM3: R^2^ = 0.04, p = 0.57).

Structural degradation was associated with increased abundances of Diptera and Turbellaria, demonstrating the euryoecious biology of respective species (*e.g.*, Chironomidae; Figure 1). On the other hand, abundances of more stenoecious Plecoptera species decreased with increased structural modification (Figure 1).

For the spring sampling event, the multiple linear regression model identified OC1 as the most significant impact variable on all biotic metrics apart from the number of individuals per m^2^, which was more affected by PAH1 and PAH2 (Table 3). In the autumn sampling event, water body structure was the most significant impact variable on individuals per m^2^ and number of taxa, while for metrics covering more sensitive species (EPT taxa and saprobic index; *e.g.*, [49]), the WW-associated contaminant factor, OC1, was the most prominent impact variable (Table 3). This might indicate a higher susceptibility of respective taxa to corresponding organic contaminants. HM1, HM3, PAH1 and average discharge are all related to Shannon and Simpson diversity. The affecting variables identified by the regression model (water body structure, OCl, HM1 and HM3) were similar to the results of the NMDS although with the NMDS we considered the whole set of recorded species. Seasonal differences in species composition between both sampling campaigns might explain differing results between spring and autumn (*e.g.*, [50]).

**Table 3:** Multiple linear regression models testing the effect of environmental parameters and contaminants on biotic response variables: the total number of individuals and taxa, Simpson and Shannon diversity, number of EPT taxa and the saprobic index.

response variable	significant impact variables									
	spring					autumn				
		p	df	F	R^2^		p	df	F	R^2^
**individuals/m^2^**	PAH2	★★	1	18.77	n.a.	water structure	★	1	5.14	n.a.
	PAH1	★★	1	8.63	n.a.	HM1	★	1	4.53	n.a.
	*full model*	★★	5,20	7.09	0.55	*full model*	n.s.	3,20	2.76	0.19
**number of taxa**	OC1	★★	1	22.02	n.a.	water structure	★★	1	9.09	n.a.
	PAH1	★	1	5.02	n.a.	average discharge	★★	1	8.47	n.a.
	HM3	★	1	4.37	n.a.	PAH1	★	1	7.39	n.a.
	*full model*	★★	4,21	10.62	0.61	*full model*	★★	3,20	5.69	0.38
**Simpson diversity**	OC1	★★	1	8.79	n.a.	HM3	★	1	5.35	n.a.
	OC2	★	1	6.05	n.a.	PAH1	★	1	4.89	n.a.
	HM1	★	1	6.19	n.a.	*full model*	n.s.	8,15	1.88	0.23
	*full model*	★★	5,20	10.48	0.65					
**Shannon diversity**	OC1	★★	1	12.30	n.a.	PAH1	★★	1	12.90	n.a.
	*full model*	★★	5,20	7.78	0.58	HM3	★★	1	8.97	n.a.
						average discharge	★	1	5.93	n.a.
						HM1	★	1	5.32	n.a.
						*full model*	★	5,18	3.37	0.34
**EPT taxa**	OC1	★★	1	10.50	n.a.	OC1	★★	1	15.82	n.a.
	*full model*	★★	4,21	4.99	0.39	water structure	★★	1	10.15	n.a.
						HM3	★★	1	9.18	n.a.
						*full model*	★★	5,18	4.68	0.44
**saprobic index**	OC1	★★	1	17.18	n.a.	OC1	★★	1	24.27	n.a.
	HM1	★	1	4.40	n.a.	HM3	★	1	5.70	n.a.
	*full model*	★★	4,21	9.07	0.56	HM1	★	1	4.86	n.a.
						*full model*	★★	6,17	7.48	0.63

Given are df-, R^2^-, F- and p-values for full models after stepwise deletion of non-significant terms (n.s.) and of significant model parameters.

★ , p<0.05;

★★ , p<0.01;

★★★ , p<0.001; n.a., not available.

**Figure 2:**
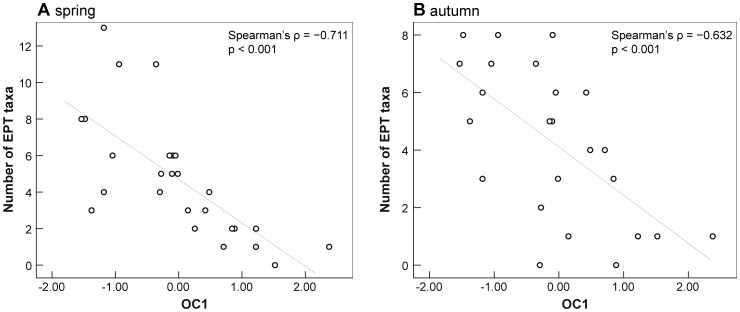
Number of EPT taxa (Ephemeroptera, Plecoptera, Trichoptera) correlating with the first component of the PCA with organic contaminants (OC1). Displayed are results for sampling campaign in spring (A) and autumn (B). Please note different scaling of y-axes in A and B.

**Figure 3:**
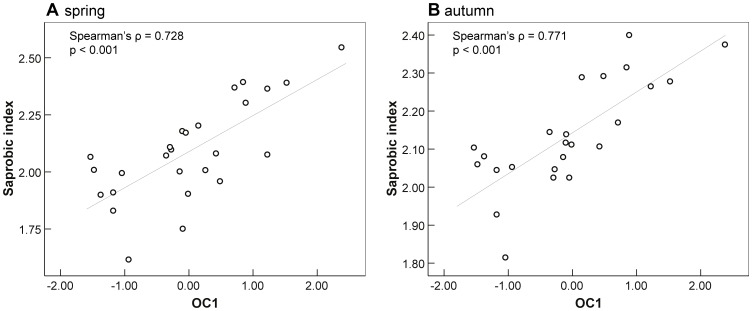
Saprobic index correlating with the first component of the PCA with organic contaminants (OC1). Displayed are results for sampling campaign in spring (A) and autumn (B). Please note different scaling of y-axes in A and B.

The correlation analyses–including macroinvertebrate taxa, biotic indices, and environmental variables–revealed consistent and highly significant correlations between organic pollutant concentration (OC1) and indices encompassing sensitive taxa. Biotic metrics like the saprobic index (Figure 2), number of EPT taxa (Figure 3), and EPTCOB taxa (Ephemeroptera, Plecoptera, Trichoptera, Coleoptera, Odonata, Bivalvia) significantly correlated with OC1 (p<0.001) revealing correlation coefficients (Spearman’s ρ) ranging from −0.63 to −0.81 (ρ = 0.73 and ρ = 0.77 in case of the saprobic index). Moreover, single genera of the orders Ephemeroptera and Coleoptera correlated significantly with OC1 (*Ephemera danica*: ρ = −0.70; *Elmis*/*Limnius*/*Haliplus*: ρ ranging from −0.45 to −0.53). Accordingly, numbers of Ephemeroptera and Coleoptera taxa and their abundances also highly negatively correlated with OC1 (p<0.001; ρ<−0.7).

## Discussion

### Key Factors Impacting Species Composition

The results of NMDS and the multiple linear regressions suggest that WW-associated organic contaminants (OC1)–alongside with structural degradation–play a pivotal role in shaping macroinvertebrate species composition in anthropogenically disturbed surface waters. The correlation analyses confirmed a deleterious relationship and indicated a reduced biodiversity of EPT taxa and Coleoptera with increasing concentrations of WW-associated trace organic contaminants. The number of EPT taxa as well as the saprobic index did not correlate with other contaminant factors that were identified as significantly affecting the macroinvertebrates in NMDS and multiple linear regression models (OC2, PAH1, HM1, HM3). This might indicate that OC1 and/or co-correlated variables were among the main negative impact factors for the macroinvertebrate communities.

Significant impact variables in the NMDS and the regression model did not necessarily have a deleterious effect. For instance, HM3 was identified as significantly affecting the species composition in the NMDS and regression analyses. HM3 represents the presence of Cd in the sediments (Table S8) suggesting a deleterious relationship due to toxic effects of Cd. However, a positive correlation of HM3 with the distance to the next WW treatment plant and a negative correlation with the WW load (p<0.01) indicated a non-causal relationship (Table S1). This is supported by predominantly low Cd concentrations of <0.9 mg/kg sediment (dry weight; apart from Mo1 with 2.4 mg/kg). Regarding factor PAH1, concentrations of PAHs exceed ‘probable effect levels’–as proposed by Macdonald and colleagues [51]–at La1 and Mo3 by up to 20 times. At these sampling sites macroinvertebrate communities might be adversely affected. However, neither NMDS nor correlation analyses were able to support a significant impact of PAHs.

Furthermore, macroinvertebrate taxa and biotic metrics were not correlated with the water body structure. This might, however, mainly be a result of the rather homogenous sampling sites in terms of structural degradation (predominantly very highly modified, Figure S5) and does not indicate that water body structure is less relevant.

The ecological quality class determined via ASTERICS did not correlate with the contaminant factors. However, ASTERICS is designed to detect general degradation that is caused by multiple factors. The pervasive structural deterioration of the sampling sites (Figure S5) might have a predominant impact on the quality class determination masking any potential relationship between quality class and pollution.

In general, the correlation analyses revealed a principle problem with identifying environmental variables affecting species assemblages. The saprobic index and the number of EPT taxa, for example, were significantly correlated (p<0.01) because many EPT taxa exhibit low tolerance towards low oxygen conditions and hence have a low saprobic rate resulting in a considerable impact on the saprobic index. As the saprobic index is a measure of O_2_ deficiencies due to decaying organic matter, impacts by insufficient O_2_ concentrations and pollutant toxicities are hardly distinguishable. In our case, OC1 was significantly correlated with O_2_ concentrations and biological oxygen demand (Table S1). In many studies low oxygen concentrations have been revealed to have a significant impact on benthic invertebrates, in particular on Ephemeroptera, Plecoptera, and Trichoptera (*e.g.*, [52]). However, our field measurements suggested good dissolved oxygen concentrations, with the bulk of measurements falling between 9.9 and 13.2 mg/L, with one exception: La1 with an average of 7.1 mg/L. Pronounced changes in aquatic species composition are not expected at concentrations >8 mg/L [53]. However, the presence and potential impact of an oxygen deficiency may have been underestimated because we did not record oxygen concentrations at night and increased nocturnal oxygen consumption by algae or submerged aquatic macrophytes can reduce oxygen saturation considerably [54].

OC1 was also associated with increased salinity (ρ = 0.93, Table S1). Salinity has been suspected to affect species composition elsewhere [53] and it is possible that the relationship observed between OCl and species assemblages was more related to salinity and not the WW-associated contaminants [55]. Again, Ephemeroptera, Plecoptera, and Trichoptera have been shown to react more sensitively to salinity than other benthic invertebrate species [55]. Therefore, a negative correlation of EPT taxa with salinity might be expected. Pond [56] documented a significant loss of mayfly taxa in Appalachian mining regions when conductivity exceeded 175 µS/cm. The average conductivity at our sampling sites ranged from 419 to 1350 µS/cm indicating a potential threat towards salinity-sensitive species. However, salinity in lowland streams is generally higher compared to streams in higher mountain ranges. Hence, lowland species should be more adapted to higher salinity levels and therefore salinity effects were expected to be less pronounced.

In the present study, salinity increase was mainly caused by the WW burden as indicated by the high correlation coefficient (ρ = 0.93) and was therefore regarded as anthropogenic disturbance. Depending on the causal factors influencing environmental salinity levels, high salt concentrations might often be accompanied by high contaminant levels (*e.g.*, in mining regions [49]). Accordingly, impacts by contaminants and salinity can hardly be distinguished. However, an indicator for WW-associated contaminants having an impact is the significant correlation of OC1 with Coleoptera genera (*Elmis*/*Limnius*/*Haliplus*: ρ ranging from −0.45 to −0.53) because they are much less salinity-sensitive than other groups of macroinvertebrate species [57].

In General, EPT taxa are more susceptible to toxicants than other groups of macroinvertebrates [58] and in particular to OCs [59]. Accordingly, a toxicity-related impact can be assumed. However, the contaminants represented by OC1 are apparently of minor ecotoxicological relevance to the EPT taxa at the quantified concentrations, as the lowest observed effect concentrations are 3–4 orders of magnitudes higher [60]–[67]. This indicates that organophosphates, synthetic musk fragrances, and DEET were not responsible for the relationship between OC1 and species composition. This is further supported by weak correlations of the TU-based factors (to account for toxic characteristics of the considered contaminants) with biota (data not shown). Additionally, the inclusion of peak concentrations (90% percentiles of measurements) did not enhance correlations. Nevertheless, our results demonstrate that compounds represented by OC1 are a good surrogate for contaminants associated with municipal WW and they may be a suitable set of markers for the identification of WW-contamination as their main entry pathway to surface waters is through the sewage system. Among OC1 pollutants, synthetic musk fragrances have previously been proposed as a marker for municipal WW by Buerge *et al.* [68]. Therefore, we assume that the basic cause for the relationship between species composition and OC1 was due to alterations of physico-chemical water parameters and/or other contaminants present in sewage effluents that were not considered in our study.

The WW-associated factor responsible for the deterioration of aquatic fauna populations–oxygen concentration, salinity or contaminants–could not be unequivocally identified with the available data. It is possible that all factors contributed to the observed impact on macroinvertebrate assemblages. Long-term observations of wastewater impacted streams may help to tease apart these factors and identify the primary factor; particularly at sites upgraded with advanced WW treatment technologies for enhanced pollutant removal [27], [69]. If an upgrade with enhanced pollutant removal technologies resulted in an increase in biodiversity, it could be unequivocally determined that the pollutants were primarily responsible for the decline in invertebrate populations.

Advanced WW treatment is supposed to be an appropriate measure to improve the ecological status of WW-impacted surface waters via contaminant degradation/removal. However, the potential benefits and adverse impacts have to be carefully evaluated to ensure that upgrading WW treatment facilities is a worthwhile exercise. For example, a potential adverse impact associated with enhanced pollutant removal technologies, like advanced oxidation processes, is the formation of hazardous transformation products. Environmental releases of these by-products may outweigh the benefits of treatment plant upgrades to include enhanced pollutant removal [70]–[73].

## Conclusion

Our study emphasizes a clear wastewater-associated impact on the ecological quality, *e.g.*, decline in biodiversity, of surface waters, despite state-of-the-art wastewater treatment with biological activated sludge. As main factor threatening the ecological quality we identified wastewater-associated contaminants– despite their minute concentrations in the ng/L range–alongside with the structural modification of the streams. Consequently, we suggest that restoration measures should aim at improving both water as well as structural quality of surface waters in order to increase their ecological quality and help to conserve freshwater biodiversity.

## Supporting Information

Figure S1 **Sampling sites characteristics of the Schwarzbach river system.**
(PDF)

Figure S2 **Sampling sites characteristics of the Modau/Sandbach river system.**
(PDF)

Figure S3 **Sampling sites characteristics of the Winkelbach river system.**
(PDF)

Figure S4 **Sampling sites characteristics of the Weschnitz river system.**
(PDF)

Figure S5 **Sampling sites characteristics.**
(PDF)

Table S1 **Environmental variables considered in the analyses and their correlations.**
(PDF)

Table S2 **List of taxa of aquatic invertebrates found during the sampling campaign in September 2005 at 24 sampling points.**
(PDF)

Table S3 **List of taxa of aquatic invertebrates found during the sampling campaign in March 2006 at 26 sampling points.**
(PDF)

Table S4 **Average concentrations of 12 organic contaminants in the water phase.**
(PDF)

Table S5 **Average concentrations of 12 heavy metals/metalloids in the sediments.**
(PDF)

Table S6 **Average concentrations of 16 polycyclic aromatic hydrocarbons in the sediments.**
(PDF)

Table S7 **Loading matrix of principle components calculated by a principle component analysis of organic contaminants listed in** **Table 2** **.**
(PDF)

Table S8 **Loading matrix of principle components calculated by a principle component analysis of heavy metals listed in** **table 2** **.**
(PDF)

Table S9 **Loading matrix of principle components calculated by a principle component analysis of polycyclic aromatic hydrocarbons listed in** **table 2** **.**
(PDF)
