# Supplementary material for: Do Contaminants Originating from State-of-the-Art Treated Wastewater Impact the Ecological Quality of Surface Waters?
Source: PLoS One. 2013 Apr 8;8(4):e60616. doi: 10.1371/journal.pone.0060616 (PMC3620539; doi:10.1371/journal.pone.0060616)
Supplement: Figure S1 — Sampling sites characteristics of the Schwarzbach river system. (PDF) [file pone.0060616.s001.pdf]

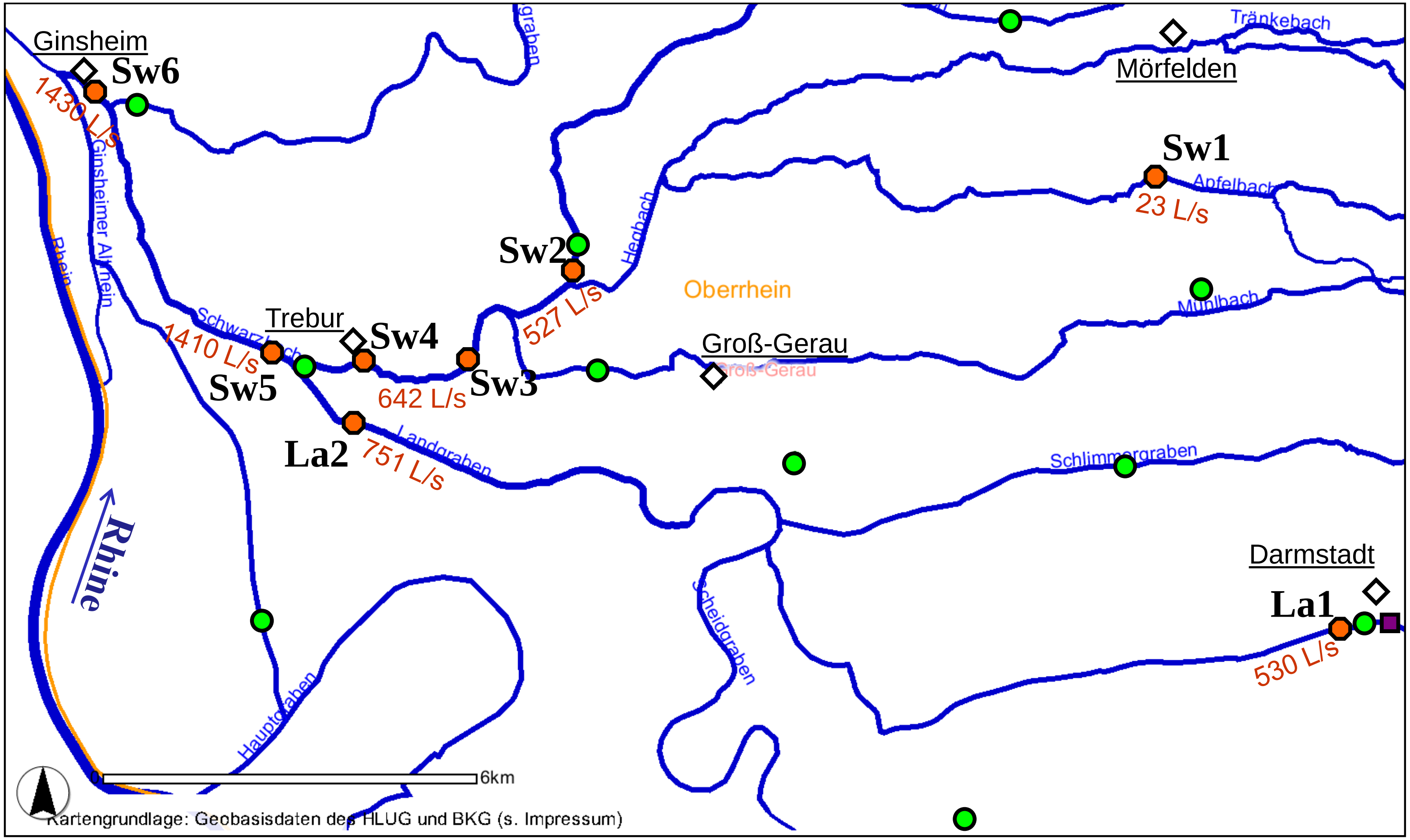

Figure S1. Sampling sites characteristics of the Schwarzbach river system. Sampling point location (orange), wastewater treatment plant discharger (green), estimated wastewater load in L/s, cities (white diamond), industrial discharger (purple square). Data based on information of the Hessian State Office for Environment and Geology [Hessisches Landesamt für Umwelt und Geologie].
