# Supplementary material for: Do Contaminants Originating from State-of-the-Art Treated Wastewater Impact the Ecological Quality of Surface Waters?
Source: PLoS One. 2013 Apr 8;8(4):e60616. doi: 10.1371/journal.pone.0060616 (PMC3620539; doi:10.1371/journal.pone.0060616)
Supplement: Figure S5 — Sampling sites characteristics. (PDF) [file pone.0060616.s005.pdf]

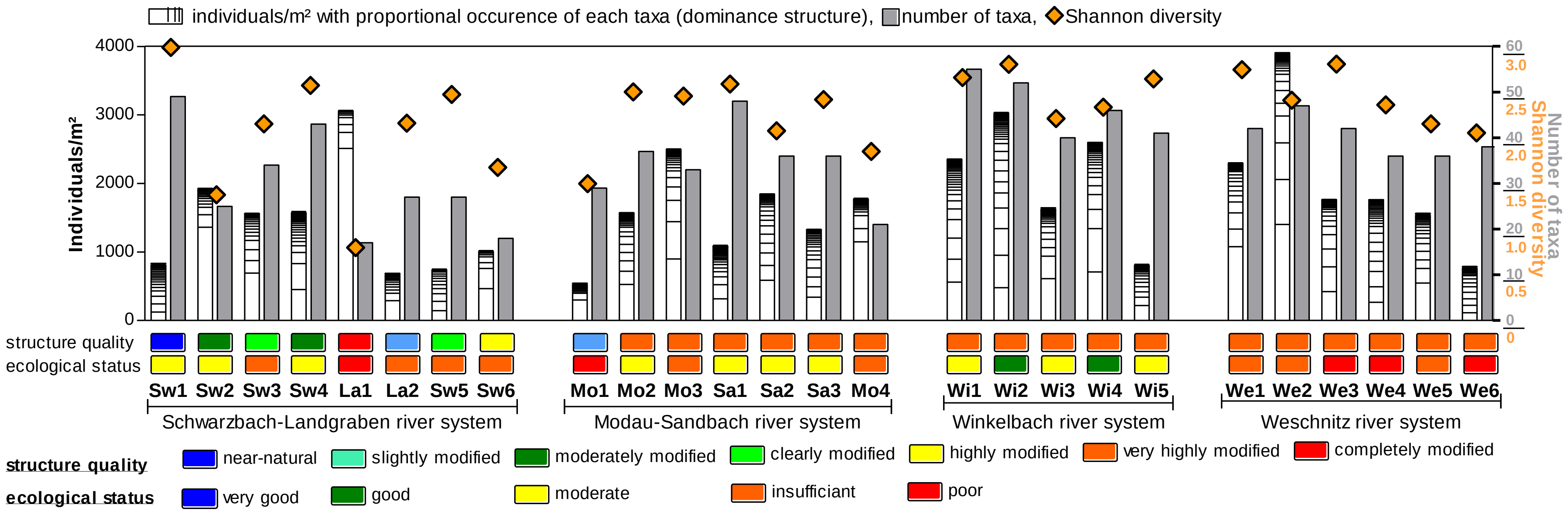

Figure S5. Sampling sites characteristics. Individuals/m² with proportional occurrence of each taxa (white columns), Number of taxa (grey columns), Shannon-Wiener diversity index (orange diamonds), structure quality, and ecological status according to ASTERICS.
