# Supplementary material for: Do Contaminants Originating from State-of-the-Art Treated Wastewater Impact the Ecological Quality of Surface Waters?
Source: PLoS One. 2013 Apr 8;8(4):e60616. doi: 10.1371/journal.pone.0060616 (PMC3620539; doi:10.1371/journal.pone.0060616)
Supplement: Table S1 — Environmental variables considered in the analyses and their correlations. (PDF) [file pone.0060616.s006.pdf]

Table S1. Environmental variables considered in the analyses and their correlations (Spearman, two-sided) with correlation factor and significance level (significance of correlations: *, p<0.05; **. p<0.01; ***, p<0.001).

	**physico-/chemical water parameter**													
	conductivity [µS/cm]	pH	flow velocity [m/s]	ammonium [mg/L]	chloride [mg/L]	phosphate [mg/L]	hardness [°dH]	carbonate hardness [°dH]	O_2 [mg/L]	BOD_5 [mg/L]	sediment mean grain size [mm]	sediment organic carbon content [%]	average discharge (L/s]	distance to the Rhine confluence [km]
conductivity [µS/cm]	1.000	-.594(**)	-0.248	.448(*)	.901(**)	.693(**)	.575(**)	.625(**)	-.691(**)	-.408(*)	-0.167	0.023	0.199	-0.331
pH	-.594(**)	1.000	0.265	-0.256	-0.386	-.536(**)	-0.108	-.393(*)	.720(**)	.488(*)	0.161	0.162	-.445(*)	-0.224
flow velocity [m/s]	-0.248	0.265	1.000	0.008	-0.247	-0.272	-0.170	-0.219	0.168	0.056	.587(**)	-0.334	0.258	0.355
ammonium [mg/L]	.448(*)	-0.256	0.008	1.000	.467(*)	.422(*)	.522(**)	0.163	-0.117	-0.188	-0.086	0.043	0.021	-0.193
chloride [mg/L]	.901(**)	-0.386	-0.247	.467(*)	1.000	.632(**)	.613(**)	.598(**)	-.479(*)	-0.320	-0.082	0.174	0.087	-.402(*)

	**WWTPs**			
	person equivalents of the upstream WWTPs	distance to the next WWTP upstream [km]	wastewater load [L/s]	wastewater load [%]
conductivity [µS/cm]	.672(**)	-.648(**)	.630(**)	.812(**)
pH	-.469(*)	.554(**)	-.609(**)	-0.327
flow velocity [m/s]	-0.077	0.082	-0.108	-.456(*)
ammonium [mg/L]	.393(*)	-0.318	0.254	.447(*)
chloride [mg/L]	.635(**)	-.621(**)	.522(**)	.864(**)

	**substrate type [% coverage]**									
	lithal (stones, grain size: 2 – 40 cm)	akal (gravel, 0.2 – 2 cm)	psammal (sand, 0.06 – 2 mm)	argyllal (fine sediemt, silt/clay, <0.06 mm)	technolithal (artificial sediment, e.g. concrete)	algae (algae covered sediment)	phytal (sediment covered with macrophytes)	xylal (woody debris)	CPOM (coarse particulate organic matter)	FPOM (fine particulate organic matter))
conductivity [µS/cm]	0.000	-0.303	0.281	0.227	0.039	0.083	-0.313	-0.038	0.189	0.262
pH	0.167	0.118	-0.249	-0.027	-0.124	-0.166	0.334	-0.097	-0.067	-0.279
flow velocity [m/s]	0.135	0.236	-0.266	0.055	0.183	0.107	-0.033	-0.079	-0.220	-0.236
ammonium [mg/L]	0.143	-0.126	0.109	0.334	0.314	-0.161	-.530(**)	0.100	.413(*)	0.384
chloride [mg/L]	0.107	-0.221	0.121	0.281	0.186	0.143	-.424(*)	-0.039	0.250	0.218

	**quality**			**contaminants**								
	water structure	ASTERICS quality class	water body type	OC1	OC2	OC3	OC4	HM1	HM2	HM3	PAH1	PAH2
conductivity [µS/cm]	-0.232	-0.296	.497(**)	.932(**)	0.033	-0.197	0.187	-0.302	-.563(**)	-.406(*)	-0.157	-.428(*)
pH	.410(*)	.406(*)	0.087	-.632(**)	-0.069	0.346	0.074	0.384	0.335	.523(**)	.413(*)	0.383
flow velocity [m/s]	.462(*)	-0.015	-0.222	-0.175	0.146	.486(*)	-0.314	0.278	.548(**)	0.201	0.349	-0.095
ammonium [mg/L]	0.059	-0.043	0.181	0.372	0.306	-0.029	0.356	0.056	-0.179	-0.379	-0.007	-0.232
chloride [mg/L]	-0.032	-0.172	.533(**)	.845(**)	0.141	-0.200	0.288	-0.113	-.511(**)	-.421(*)	0.023	-0.281

phosphate [mg/L]	hardness [°dH]	carbonate hardness [°dH]	O_2 [mg/L]	BOD_5 [mg/L]	sediment mean grain size [mm]	sediment organic carbon [%]	average discharge (L/s)	distance to the Rhine [km]	PEs of the upstream WWTPs	km to the next WWTP upstream
.693(**)	.575(**)	.625(**)	-.691(**)	-.408(*)	-0.167	0.023	0.199	-0.331	.672(**)	-.648(**)
-.536(**)	-0.108	-.393(*)	.720(**)	.488(*)	0.161	0.162	-.445(*)	-0.224	-.469(*)	.554(**)
-0.272	-0.170	-0.219	0.168	0.056	.587(**)	-0.334	0.258	0.355	-0.077	0.082
.422(*)	.522(**)	0.163	-0.117	-0.188	-0.086	0.043	0.021	-0.193	.393(*)	-0.318
.632(**)	.613(**)	.598(**)	-.479(*)	-0.320	-0.082	0.174	0.087	-.402(*)	.635(**)	-.621(**)
1.000	0.375	.396(*)	-0.366	-.469(*)	-0.107	-0.048	0.003	-0.233	0.241	-.505(**)
0.375	1.000	.431(*)	-0.188	-0.224	-0.288	0.160	-0.380	-.500(**)	0.260	-0.332
.396(*)	.431(*)	1.000	-.562(**)	-0.285	-0.219	0.228	0.270	-0.043	.583(**)	-.427(*)
-0.366	-0.188	-.562(**)	1.000	.458(*)	0.254	0.099	-0.338	-0.183	-.493(*)	.471(*)
-.469(*)	-0.224	-0.285	.458(*)	1.000	0.155	0.219	0.154	-0.110	-0.060	.400(*)
-0.107	-0.288	-0.219	0.254	0.155	1.000	-.577(**)	0.228	0.243	0.031	-0.057
-0.048	0.160	0.228	0.099	0.219	-.577(**)	1.000	-0.060	-0.244	-0.004	-0.032
0.003	-0.380	0.270	-0.338	0.154	0.228	-0.060	1.000	0.305	.649(**)	-.397(*)
-0.233	-.500(**)	-0.043	-0.183	-0.110	0.243	-0.244	0.305	1.000	-0.226	0.145
0.241	0.260	.583(**)	-.493(*)	-0.060	0.031	-0.004	.649(**)	-0.226	1.000	-.599(**)
-.505(**)	-0.332	-.427(*)	.471(*)	.400(*)	-0.057	-0.032	-.397(*)	0.145	-.599(**)	1.000
0.339	0.013	.541(**)	-.572(**)	-0.052	-0.013	-0.014	.809(**)	-0.061	.907(**)	-.605(**)
.715(**)	.507(**)	.512(**)	-0.304	-0.216	-0.231	0.199	0.081	-.549(**)	.566(**)	-.572(**)
-0.015	0.167	0.154	0.320	-0.091	0.157	0.022	-0.036	-0.109	0.057	-0.168
-0.287	-0.296	-0.198	0.124	0.085	.544(**)	-0.348	0.011	0.266	-0.053	0.342
0.223	0.032	0.036	-0.327	0.018	-0.361	0.070	0.116	-0.053	0.109	-0.293
0.328	0.284	-0.085	0.040	0.027	0.096	0.093	-0.013	-0.280	0.120	-0.227
0.078	0.274	0.205	0.110	-0.079	0.164	0.297	0.050	0.183	0.015	-0.267
-0.099	-0.097	0.218	-0.208	0.055	0.385	-0.121	0.379	0.059	.408(*)	-0.186
-0.253	-0.050	-0.053	0.276	0.129	-0.255	0.082	-0.321	-0.241	-0.387	.530(**)
-0.124	0.123	-0.123	-0.096	-0.299	-0.139	-0.280	-0.308	0.183	-0.106	0.212
0.339	0.299	-0.096	0.158	-0.300	-0.033	-0.078	-0.367	-0.273	-0.004	-0.004
.484(*)	0.248	-0.017	-0.178	-0.337	-0.175	-0.281	-0.158	-0.106	0.000	-0.239
-.467(*)	0.056	-0.184	0.305	0.153	.422(*)	0.123	0.016	-0.025	0.014	-0.043
-0.211	0.056	-0.273	0.325	0.042	-0.210	0.200	-0.381	-.490(*)	-0.265	0.331
.393(*)	.512(**)	0.281	-0.238	-0.329	-.399(*)	0.377	-0.369	-.591(**)	0.051	-0.287
.647(**)	.448(*)	.597(**)	-.656(**)	-.390(*)	-0.152	0.060	0.363	-0.215	.681(**)	-.748(**)
-0.117	-0.023	-0.284	0.022	-0.117	.390(*)	-0.324	0.057	-0.004	0.292	-0.166
-0.228	0.346	-0.227	0.279	0.005	0.232	-0.110	-0.310	-0.055	-0.326	0.062
0.360	.492(*)	-0.028	0.069	-.512(**)	-0.283	0.060	-.616(**)	-.496(**)	-0.090	-0.119
-0.341	0.052	-0.014	.433(*)	0.209	0.137	0.347	-0.104	0.256	-0.177	0.194
-.445(*)	-.513(**)	-.526(**)	0.382	0.325	.573(**)	-.472(*)	0.275	0.318	-0.105	0.279
-0.363	-0.273	-0.327	0.213	0.097	-0.108	0.030	-0.283	0.058	-.533(**)	.481(*)
-0.124	0.137	0.064	0.275	-0.127	0.105	0.282	-0.313	0.021	-0.252	0.132
-0.245	-0.150	-0.107	0.341	0.290	-0.094	.598(**)	-0.236	0.090	-.444(*)	0.151

wastewater load [L/s]	wastewater load [%]	lithal	akal	psammal	argyllal	technolithal	algae	phytal	xylal	CPOM
.630(**)	.812(**)	0.000	-0.303	0.281	0.227	0.039	0.083	-0.313	-0.038	0.189
-.609(**)	-0.327	0.167	0.118	-0.249	-0.027	-0.124	-0.166	0.334	-0.097	-0.067
-0.108	-.456(*)	0.135	0.236	-0.266	0.055	0.183	0.107	-0.033	-0.079	-0.220
0.254	.447(*)	0.143	-0.126	0.109	0.334	0.314	-0.161	-.530(**)	0.100	.413(*)
.522(**)	.864(**)	0.107	-0.221	0.121	0.281	0.186	0.143	-.424(*)	-0.039	0.250
0.339	.715(**)	-0.015	-0.287	0.223	0.328	0.078	-0.099	-0.253	-0.124	0.339
0.013	.507(**)	0.167	-0.296	0.032	0.284	0.274	-0.097	-0.050	0.123	0.299
.541(**)	.512(**)	0.154	-0.198	0.036	-0.085	0.205	0.218	-0.053	-0.123	-0.096
-.572(**)	-0.304	0.320	0.124	-0.327	0.040	0.110	-0.208	0.276	-0.096	0.158
-0.052	-0.216	-0.091	0.085	0.018	0.027	-0.079	0.055	0.129	-0.299	-0.300
-0.013	-0.231	0.157	.544(**)	-0.361	0.096	0.164	0.385	-0.255	-0.139	-0.033
-0.014	0.199	0.022	-0.348	0.070	0.093	0.297	-0.121	0.082	-0.280	-0.078
.809(**)	0.081	-0.036	0.011	0.116	-0.013	0.050	0.379	-0.321	-0.308	-0.367
-0.061	-.549(**)	-0.109	0.266	-0.053	-0.280	0.183	0.059	-0.241	0.183	-0.273
.907(**)	.566(**)	0.057	-0.053	0.109	0.120	0.015	.408(*)	-0.387	-0.106	-0.004
-.605(**)	-.572(**)	-0.168	0.342	-0.293	-0.227	-0.267	-0.186	.530(**)	0.212	-0.004
1.000	.552(**)	-0.085	-0.156	0.293	0.040	-0.025	0.328	-.415(*)	-0.183	-0.118
.552(**)	1.000	0.143	-.406(*)	0.270	0.147	0.067	-0.043	-0.338	-0.173	0.218
-0.085	0.143	1.000	-0.363	-0.271	-0.247	.409(*)	-0.123	0.049	0.069	0.205
-0.156	-.406(*)	-0.363	1.000	-.620(**)	0.043	-0.094	.525(**)	-0.011	0.000	-0.117
0.293	0.270	-0.271	-.620(**)	1.000	0.134	-0.254	-.525(**)	-0.221	-0.048	-0.082
0.040	0.147	-0.247	0.043	0.134	1.000	-0.072	-0.131	-0.198	-0.058	0.327
-0.025	0.067	.409(*)	-0.094	-0.254	-0.072	1.000	0.039	-0.356	-0.104	0.039
0.328	-0.043	-0.123	.525(**)	-.525(**)	-0.131	0.039	1.000	-0.083	-0.189	-0.073
-.415(*)	-0.338	0.049	-0.011	-0.221	-0.198	-0.356	-0.083	1.000	-0.031	-0.104
-0.183	-0.173	0.069	0.000	-0.048	-0.058	-0.104	-0.189	-0.031	1.000	.531(**)
-0.118	0.218	0.205	-0.117	-0.082	0.327	0.039	-0.073	-0.104	.531(**)	1.000
0.037	0.357	-0.163	-0.017	0.202	0.285	-0.257	-0.343	-0.256	0.103	0.043
-0.200	-0.246	0.074	.432(*)	-.459(*)	0.110	0.381	0.337	-0.141	-0.204	-0.235
-0.365	-0.093	0.110	0.038	-0.319	-0.071	0.053	-0.124	0.316	-0.103	-0.057
-0.021	.547(**)	-0.048	-0.373	0.190	0.132	0.018	-0.184	0.073	-0.178	0.027
.725(**)	.818(**)	0.015	-.398(*)	0.360	0.093	0.144	0.071	-.420(*)	-0.135	0.030
0.103	-0.005	-0.111	.609(**)	-0.374	0.093	-0.023	0.386	-.443(*)	0.135	0.101
-.514(**)	-0.346	0.238	0.063	-0.269	0.227	0.211	-0.132	0.249	-0.058	-0.164
-0.261	0.318	0.017	0.005	-0.202	0.253	-0.015	-0.101	0.010	0.308	.569(**)
-0.311	-0.204	0.212	0.155	-0.245	-0.120	.496(**)	-0.045	-0.078	-0.308	-0.217
-0.111	-.546(**)	-0.105	.532(**)	-0.336	0.147	-0.241	0.275	-0.072	-0.019	-0.106
-.535(**)	-0.380	0.132	-0.021	-0.196	-0.227	-0.317	-0.190	0.384	-0.173	-0.282
-0.333	-0.100	0.251	-0.027	-0.206	-0.120	.450(*)	-0.166	-0.019	0.000	0.039
-.419(*)	-0.266	-0.006	-0.068	0.046	-0.067	0.278	-0.021	0.077	-.423(*)	-0.192

FPOM	0.262	-0.279	-0.236	0.384	0.218	.484(*)	0.248	-0.017	-0.178	-0.337	-0.175	-0.281	-0.158	-0.106	0.000	-0.239	0.037	0.357	-0.163	-0.017	0.202	0.285	-0.257	-0.343	-0.256	0.103	0.043	1.000	-0.283	-0.003	0.145	0.234	0.219	-0.035	.428(*)	-0.352	0.002	-0.042	-0.375	-.424(*)
water structure	-0.232	.410(*)	.462(*)	0.059	-0.032	-.467(*)	0.056	-0.184	0.305	0.153	.422(*)	0.123	0.016	-0.025	0.014	-0.043	-0.200	-0.246	0.074	.432(*)	-.459(*)	0.110	0.381	0.337	-0.141	-0.204	-0.235	-0.283	1.000	0.234	0.123	-0.197	.450(*)	.508(**)	0.034	.508(**)	0.308	0.035	0.314	0.225
ASTERICS quality class	-0.296	.406(*)	-0.015	-0.043	-0.172	-0.211	0.056	-0.273	0.325	0.042	-0.210	0.200	-0.381	-.490(*)	-0.265	0.331	-0.365	-0.093	0.110	0.038	-0.319	-0.071	0.053	-0.124	0.316	-0.103	-0.057	-0.003	0.234	1.000	0.342	-0.344	0.030	0.211	0.387	-0.038	-0.003	.400(*)	0.237	0.083
water body type	.497(**)	0.087	-0.222	0.181	.533(**)	.393(*)	.512(**)	0.281	-0.238	-0.329	-.399(*)	0.377	-0.369	-.591(**)	0.051	-0.287	-0.021	.547(**)	-0.048	-0.373	0.190	0.132	0.018	-0.184	0.073	-0.178	0.027	0.145	0.123	0.342	1.000	.445(*)	-0.161	0.090	.530(**)	-0.117	-.606(**)	0.051	0.204	0.045
OC1	.932(**)	-.632(**)	-0.175	0.372	.845(**)	.647(**)	.448(*)	.597(**)	-.656(**)	-.390(*)	-0.152	0.060	0.363	-0.215	.681(**)	-.748(**)	.725(**)	.818(**)	0.015	-.398(*)	0.360	0.093	0.144	0.071	-.420(*)	-0.135	0.030	0.234	-0.197	-0.344	.445(*)	1.000	0.007	-0.256	0.048	-0.216	-.525(**)	-.441(*)	-0.108	-0.380
OC2	0.033	-0.069	0.146	0.306	0.141	-0.117	-0.023	-0.284	0.022	-0.117	.390(*)	-0.324	0.057	-0.004	0.292	-0.166	0.103	-0.005	-0.111	.609(**)	-0.374	0.093	-0.023	0.386	-.443(*)	0.135	0.101	0.219	.450(*)	0.030	-0.161	0.007	1.000	-0.008	0.276	0.082	.399(*)	-0.178	-0.088	-0.313
OC3	-0.197	0.346	.486(*)	-0.029	-0.200	-0.228	0.346	-0.227	0.279	0.005	0.232	-0.110	-0.310	-0.055	-0.326	0.062	-.514(**)	-0.346	0.238	0.063	-0.269	0.227	0.211	-0.132	0.249	-0.058	-0.164	-0.035	.508(**)	0.211	0.090	-0.256	-0.008	1.000	0.050	0.325	0.121	0.319	0.315	0.150
OC4	0.187	0.074	-0.314	0.356	0.288	0.360	.492(*)	-0.028	0.069	-.512(**)	-0.283	0.060	-.616(**)	-.496(**)	-0.090	-0.119	-0.261	0.318	0.017	0.005	-0.202	0.253	-0.015	-0.101	0.010	0.308	.569(**)	.428(*)	0.034	0.387	.530(**)	0.048	0.276	0.050	1.000	-0.223	-0.211	-0.104	0.075	-0.173
HM1	-0.302	0.384	0.278	0.056	-0.113	-0.341	0.052	-0.014	.433(*)	0.209	0.137	0.347	-0.104	0.256	-0.177	0.194	-0.311	-0.204	0.212	0.155	-0.245	-0.120	.496(**)	-0.045	-0.078	-0.308	-0.217	-0.352	.508(**)	-0.038	-0.117	-0.216	0.082	0.325	-0.223	1.000	0.048	0.159	.552(**)	.553(**)

HM2	-.563(**)	0.335	.548(**)	-0.179	-.511(**)	-.445(*)	-.513(**)	-.526(**)	0.382	0.325	.573(**)	-.472(*)	0.275	0.318
HM3	-.406(*)	.523(**)	0.201	-0.379	-.421(*)	-0.363	-0.273	-0.327	0.213	0.097	-0.108	0.030	-0.283	0.058
PAH1	-0.157	.413(*)	0.349	-0.007	0.023	-0.124	0.137	0.064	0.275	-0.127	0.105	0.282	-0.313	0.021
PAH2	-.428(*)	0.383	-0.095	-0.232	-0.281	-0.245	-0.150	-0.107	0.341	0.290	-0.094	.598(**)	-0.236	0.090

HM2	-0.105	0.279	-0.111	-.546(**)
HM3	-.533(**)	.481(*)	-.535(**)	-0.380
PAH1	-0.252	0.132	-0.333	-0.100
PAH2	-.444(*)	0.151	-.419(*)	-0.266

HM2	-0.105	.532(**)	-0.336	0.147	-0.241	0.275	-0.072	-0.019	-0.106	0.002
HM3	0.132	-0.021	-0.196	-0.227	-0.317	-0.190	0.384	-0.173	-0.282	-0.042
PAH1	0.251	-0.027	-0.206	-0.120	.450(*)	-0.166	-0.019	0.000	0.039	-0.375
PAH2	-0.006	-0.068	0.046	-0.067	0.278	-0.021	0.077	-.423(*)	-0.192	-.424(*)

HM2	0.308	-0.003	-.606(**)
HM3	0.035	.400(*)	0.051
PAH1	0.314	0.237	0.204
PAH2	0.225	0.083	0.045

HM2	-.525(**)	.399(*)	0.121	-0.211	0.048	1.000	0.221	-0.223	-0.179
HM3	-.441(*)	-0.178	0.319	-0.104	0.159	0.221	1.000	0.172	0.244
PAH1	-0.108	-0.088	0.315	0.075	.552(**)	-0.223	0.172	1.000	.445(*)
PAH2	-0.380	-0.313	0.150	-0.173	.553(**)	-0.179	0.244	.445(*)	1.000
