# Supplementary material for: Do Contaminants Originating from State-of-the-Art Treated Wastewater Impact the Ecological Quality of Surface Waters?
Source: PLoS One. 2013 Apr 8;8(4):e60616. doi: 10.1371/journal.pone.0060616 (PMC3620539; doi:10.1371/journal.pone.0060616)
Supplement: Table S2 — List of taxa of aquatic invertebrates found during the sampling campaign in September 2005 at 24 sampling points. (PDF) [file pone.0060616.s007.pdf]

Table S2. List of taxa of aquatic invertebrates found during the sampling campaign in September 2005 at 24 sampling points (no biota sampling was possible at Mo4 and Sw6 due to too high water levels).

Class	Order	Family	Taxon	La 1	La 2	Mo 1	Mo 2	Mo 3	Mo4	Sa 1	Sa 2	Sa 3	Sw 1	Sw 2	Sw 3	SW 4	Sw 5	Sw6	We 1	We 2	We 3	We 4	We 5	We 6	Wi 1	Wi 2	Wi 3	Wi 4	Wi 5
Cestoda		indet.	*Cestoda Gen. sp.*	0	0	0	0	0		0	0	0	0	0	0	0	0		0	0	0	0	0	0	0	0	0	0	0
Nematoda		indet.	*Nematoda Gen. sp.*	2	1	0	0	0		0	0	0	0	0	0	0	4		0	0	0	0	0	0	15	0	0	0	0
Turbellaria	Tricladida	Dendrocoelidae	*Dendrocoelum lacteum*	0	0	0	0	0		0	0	0	0	0	0	0	0		0	0	0	0	0	0	0	0	0	0	0
		Dugesiidae	*Dugesia sp.*	0	2	0	0	0		0	0	0	0	0	0	0	0		0	0	0	0	0	0	0	0	26	0	0
		Planariidae	*Planaria sp.*	0	0	0	0	0		0	0	0	2	0	0	0	0		0	0	0	0	12	0	0	0	0	0	0
Bivalvia	Eulamelli-branchiata	Corbiculidae	*Corbicula fluminea*	0	14	1	0	20		0	0	0	0	0	50	72	34		0	0	0	0	0	100	0	0	0	515	57
		Sphaeriidae	*Musculium transversum*	0	0	0	0	0		0	0	0	0	0	0	12	0		0	0	0	0	0	0	0	0	0	0	0
			Pisidium sp.	0	23	7	27	195		0	0	24	0	0	0	42	23		0	6	0	0	12	0	12	0	0	35	0
			Sphaerium corneum	0	0	8	0	45		2	3	0	0	0	0	0	0		7	0	35	0	36	0	0	0	0	0	0
			Sphaerium rivicola	0	0	0	0	0		0	0	0	0	0	0	0	0		0	0	0	0	0	0	0	0	0	0	0
			Sphaerium sp.	0	1	2	9	5		0	0	0	0	0	1	12	0		0	0	0	0	0	0	0	0	0	0	0
		Unionidae	*Unio pictorum ssp.*	0	0	0	0	0		0	0	0	0	0	0	0	0		0	0	0	0	0	0	0	0	0	0	0
Gastropoda	Prosobranchia	Bithyniidae	*Bithynia tentaculata*	0	0	0	0	0		0	0	0	0	0	0	0	4		0	0	0	0	0	0	0	0	0	0	0
		Hydrobiidae	*Potamopyrgus antipodarum*	0	12	0	0	50		0	655	44	0	0	0	18	109		0	0	0	0	0	0	147	0	0	0	2
	Basommatophora	Ancylidae	*Ancylus fluviatilis*	0	0	8	27	0		20	3	4	0	0	0	0	0		7	150	0	0	0	0	0	0	0	0	0
		Lymnaeidae	*Galba truncatula*	0	0	0	0	0		0	0	0	0	0	0	0	0		0	0	0	0	0	0	0	0	0	0	0
			Lymnaeidae Gen. sp.	0	0	0	6	0		0	0	0	0	0	0	0	0		0	0	0	0	0	0	0	0	0	0	0
			Radix auricularia	0	0	0	0	0		0	0	0	0	0	0	0	0		0	0	0	0	0	0	0	7	4	0	0
			Radix balthica	0	0	0	0	0		1	0	0	0	0	0	0	0		0	0	0	0	0	0	12	0	0	0	0
			Radix sp.	0	0	0	0	0		0	0	0	0	0	0	0	0		0	0	0	0	0	0	0	17	0	0	0
			Stagnicola sp.	0	0	0	0	0		0	0	0	0	0	0	0	0		0	0	0	0	0	0	0	0	0	0	0
		Physidae	*Physa fontinalis*	0	0	0	0	0		0	13	6	0	0	0	0	0		0	0	0	0	0	0	0	0	0	0	0
			Physella sp.	0	0	0	0	0		0	0	0	0	0	0	0	0		0	0	0	0	0	0	0	0	0	0	0
			Physidae Gen. sp.	0	0	0	0	0		0	0	0	0	0	0	0	0		0	0	0	0	0	0	0	0	0	0	0
		Planorbidae	*Bathyomphalus contortus*	0	0	0	0	0		0	0	0	0	0	0	0	0		0	0	0	0	0	0	0	0	0	0	0

			Gyraulus sp.	0	0	0	0	5	0	0	0	0	0	0	0	0	0	0	0	0	0	0	0	0	0	0	0
			Planorbidae Gen. sp.	0	0	0	0	0	1	0	0	0	0	0	0	0	0	0	0	0	0	0	0	0	0	0	0
Annelida	Oligochaeta	indet.	*Oligochaeta Gen. sp.*	23	7	11	42	40	15	113	20	1	40	0	54	23	10	24	30	23	42	25	21	0	0	115	25
	Hirudinea	Erpobdellidae	*Erpobdella octoculata*	0	1	0	51	10	2	0	0	0	0	2	0	4	17	6	5	0	0	0	15	0	0	0	0
		Glossiphoniidae	*Glossiphonia complanata*	0	0	0	0	0	0	0	0	0	0	0	0	0	0	0	0	0	0	0	0	0	0	0	0
			Glossiphonia concolor	0	0	0	6	0	0	0	0	0	0	0	0	0	0	0	0	0	0	0	0	0	0	0	0
			Glossiphonia verrucata	0	0	0	0	0	0	0	0	0	0	0	0	0	0	0	0	0	0	0	3	0	0	0	0
			Helobdella stagnalis	0	1	0	39	40	1	0	0	0	0	1	6	79	17	0	5	4	0	0	0	3	0	10	13
		Piscicolidae	*Piscicolidae Gen. sp.*	0	0	0	0	0	0	0	0	1	0	0	0	0	0	0	0	0	0	0	0	0	0	0	0
Crustacea	Amphipoda	Crangonycitidae	*Crangonyx pseudogracilis*	0	0	0	0	0	0	0	0	0	0	0	0	0	0	0	0	0	0	0	0	0	0	0	0
		Gammaridae	*Dikerogammarus sp.*	0	0	0	0	0	0	0	0	0	0	0	0	0	0	0	0	0	0	0	0	0	0	0	3
			Dikerogammarus villosus	0	0	0	0	0	0	0	0	0	0	0	0	0	0	0	0	0	0	0	0	0	0	0	22
			Gammarus fossarum	0	0	0	0	0	0	0	0	1	0	0	0	0	23	0	0	0	0	0	0	0	0	0	0
			Gammarus lacustris	0	0	0	0	0	0	0	0	0	0	0	0	0	0	0	0	0	0	0	0	0	0	0	0
			Gammarus pulex	0	0	746	108	0	6	0	0	0	0	0	0	0	0	0	0	0	0	0	0	0	0	0	0
			Gammarus roeselii	2	0	15	219	5	30	0	0	12	1795	0	126	731	40	0	30	15	12	0	0	0	0	0	0
			Gammarus sp.	0	0	0	60	5	13	0	0	0	95	121	12	30	0	0	0	0	0	0	0	3	0	0	0
			Gammarus tigrinus	0	22	0	0	0	0	0	0	0	0	82	0	4	0	0	0	0	0	0	0	0	0	0	0
	Isopoda	Asellidae	*Asellus aquaticus*	2	13	0	0	10	1	3	12	0	10	10	24	221	10	6	30	4	18	0	0	27	13	10	0
Insecta	Coleoptera	Dytiscidae	*Platambus maculatus Lv.*	0	0	0	0	0	0	0	0	0	0	0	0	0	0	0	0	0	0	0	0	0	0	0	0
		Elmidae	*Elmis aenea/maugetii/rietscheli/rioloides Ad.*	0	0	0	0	15	0	0	0	0	0	0	12	0	3	0	0	4	12	0	3	10	0	0	2
			Elmis latreillei Lv.	0	0	0	0	0	0	0	0	0	0	0	0	0	0	0	0	0	0	0	0	0	0	0	0
			Elmis maugetii Lv.	0	0	0	0	0	0	0	0	0	0	0	0	0	0	0	0	0	0	0	0	0	0	0	0
			Elmis rietscheli Lv.	0	0	0	0	0	0	0	0	0	0	0	0	0	0	0	0	0	0	0	0	0	0	0	0
			Elmis rioloides Lv.	0	0	0	0	0	0	0	0	0	0	0	0	0	0	0	0	0	0	0	0	0	0	0	0
			Elmis sp. Lv.	0	5	0	9	0	21	10	22	1	0	0	30	0	217	42	80	8	24	45	18	37	47	35	106
			Limnius muelleri	0	0	0	0	0	0	0	0	0	0	0	0	0	0	6	0	0	0	0	0	0	0	0	0
			Limnius volckmari Ad.	0	0	0	0	0	0	0	0	0	0	0	0	0	0	0	0	0	0	0	6	0	0	0	0
			Limnius volckmari Lv.	0	0	2	0	0	0	0	0	1	0	0	0	0	67	24	85	4	12	0	60	7	0	0	3
			Oulimnius sp. Ad.	0	0	0	0	0	0	0	0	0	0	0	0	0	0	0	0	0	0	0	0	0	4	0	3

		Oulimnius tuberculatus Lv.	0	13	1	0	15	0	13	32	0	0	2	24	0	17	252	300	49	240	10	12	56	107	20	150
	Georissidae	*Georissus sp. Ad.*	0	0	0	0	0	1	0	0	0	0	0	0	0	0	0	0	0	0	0	0	0	0	0	0
	Gyrinidae	*Gyrinidae Gen. sp. Lv.*	0	0	0	0	0	0	0	0	0	0	0	0	0	0	0	0	0	0	0	3	0	0	0	0
		Orectochilus villosus Lv.	0	0	0	0	0	0	0	0	0	0	0	0	0	0	0	0	0	0	0	3	23	0	0	0
	Haliplidae	*Haliplus (Haliplus) sp. Ad.*	0	0	0	0	0	0	0	0	0	0	0	0	0	0	0	0	0	0	0	0	0	9	0	0
		Haliplus (Liaphlus) sp. Ad.	0	0	0	0	0	0	0	0	0	0	0	0	0	0	0	0	0	0	0	0	0	4	0	0
		Haliplus lineatocollis Lv.	0	0	0	0	0	0	0	0	0	0	0	12	4	0	0	0	0	0	0	27	7	17	10	0
		Haliplus sp. Lv.	0	1	0	0	0	0	0	2	0	0	0	12	0	0	36	0	4	12	0	36	33	47	40	5
	Hydrophilidae	*Laccobius sp. Lv.*	2	0	0	0	0	0	0	0	0	0	0	0	0	0	0	0	0	0	0	0	0	4	0	0
	indet.	*Coleoptera Gen. sp. Lv.*	0	0	0	0	0	0	0	0	0	0	0	0	0	0	0	0	0	0	0	0	0	0	0	0
	Psephenidae	*Eubria palustris Lv.*	0	0	0	0	0	0	0	0	0	0	0	0	0	0	0	0	0	0	0	0	0	0	0	0
Diptera	Ceratopogonidae	*Ceratopogoninae/Palpomyiinae Gen. sp.*	2	0	0	18	20	1	3	2	0	0	0	0	0	10	0	15	4	6	0	3	3	0	5	0
	Chironomidae	*Chironominae Gen. sp.*	8	7	14	120	125	11	70	124	0	40	5	66	0	117	90	170	60	396	115	21	60	26	0	14
		Chironomini Gen. sp.	33	7	2	261	210	10	23	180	0	0	1	120	15	223	414	160	49	678	80	63	40	13	30	9
		Tanytarsini Gen. sp.	3	27	53	12	910	13	68	224	5	20	1	864	109	183	1080	255	158	1860	1430	252	183	913	400	73
		Prodiamesa olivacea	0	5	14	114	15	5	13	6	1	5	0	12	4	27	0	0	0	0	0	12	0	0	0	0
		Tanypodinae Gen. sp.	15	2	0	0	125	3	45	12	0	5	4	42	11	0	0	30	8	0	0	69	83	154	30	6
		Orthocladiinae Gen. sp.	303	3	11	15	0	35	15	16	0	0	0	0	30	147	216	185	90	168	0	117	400	163	1020	71
	Culicidae	*Culicidae Gen. sp.*	0	0	0	0	0	0	0	0	0	0	0	6	0	0	0	0	0	0	0	0	0	0	0	0
	Dolichopodidae	*Dolichopodidae Gen. sp.*	0	0	0	0	0	0	0	0	0	0	0	0	0	0	0	0	0	0	0	0	0	0	0	0
	Empididae	*Chelifera sp.*	0	0	0	0	0	0	0	0	0	0	0	0	0	0	0	0	0	0	0	0	0	0	0	0
		Clinocerinae Gen. sp.	0	0	0	0	0	0	0	0	0	0	0	0	0	0	0	0	0	0	0	0	0	0	0	0
		Hemerodromia sp.	0	0	0	0	0	0	0	0	0	0	0	0	0	0	0	0	0	0	0	0	0	0	0	0
	indet.	*Diptera Gen. sp.*	0	0	0	0	0	0	0	0	1	0	0	0	0	0	0	0	34	0	0	0	0	0	0	0
	Limoniidae/Pedic-iidae	*Dicranota sp.*	0	0	0	0	0	0	28	0	0	0	0	0	0	0	90	20	0	6	0	0	17	0	5	0
		Limoniidae Gen. sp.	0	0	0	0	0	0	0	0	0	0	0	0	0	0	0	0	0	0	0	0	0	0	0	0
	Muscidae	*Lispe sp.*	0	0	0	0	0	0	3	0	0	0	0	0	0	0	0	0	0	0	0	18	20	0	0	0
	Psychodidae	*Psychodidae Gen. sp.*	0	0	0	0	0	1	0	0	0	0	0	0	0	0	0	0	0	0	0	0	0	0	0	0
	Ptychopteridae	*Ptychoptera sp.*	0	0	0	0	0	0	0	0	0	0	0	0	0	0	0	0	0	0	0	0	0	0	0	0

	Rhagionidae	*Chrysopilus sp.*	0	0	0	0	0	1	0	0	0	0	0	0	0	0	0	0	0	0	0	0	0	0	0	0
	Sciomyzidae	*Sciomyzidae Gen. sp.*	0	0	0	0	0	0	0	0	0	0	0	0	0	0	0	0	0	0	0	0	0	0	0	0
	Simuliidae	*Simulium sp.*	0	0	3	0	0	0	0	0	0	0	0	0	0	0	18	0	0	36	0	0	0	0	0	0
	Stratiomyiidae	*Stratiomyiidae Gen. sp.*	0	0	0	0	0	0	0	0	0	0	0	0	0	0	0	0	0	0	0	0	0	0	5	0
	Tabanidae	*Chrysops sp.*	0	0	0	0	35	0	0	0	1	0	0	0	0	3	0	0	0	0	0	0	0	0	0	0
		Tabanidae Gen. sp.	0	0	0	0	0	0	0	0	0	0	0	0	0	0	0	0	0	0	0	12	3	0	0	0
	Tipulidae	*Tipula sp.*	3	0	0	0	0	0	0	0	0	0	0	0	0	0	0	0	0	0	0	0	0	0	0	0
Ephemeroptera	Baetidae	*Baetis rhodani*	0	0	0	0	0	0	0	0	0	0	0	0	0	20	0	0	0	0	0	0	0	0	0	0
		Baetis sp.	0	0	31	9	0	1	3	0	0	0	0	0	8	83	6	10	0	0	0	9	0	0	10	0
		Cloeon dipterum	0	0	0	0	0	0	0	0	0	0	0	0	0	0	0	0	0	0	0	0	0	0	0	0
	Caneidae	*Caenis pusilla*	0	0	0	0	0	0	0	0	0	0	0	0	0	0	0	0	0	0	0	0	0	0	0	0
		Caenis sp.	0	0	0	0	15	0	8	16	0	0	0	6	4	0	6	5	0	0	0	0	43	17	25	17
	Ephemerellidae	*Ephemerella sp.*	0	0	1	0	0	0	0	0	1	0	0	0	0	10	48	55	11	24	0	90	10	0	0	0
	Ephemeridae	*Ephemera danica*	0	0	0	0	0	10	0	0	2	0	0	0	0	0	0	0	0	0	0	0	0	4	0	21
	Heptageniidae	*Heptagenia sulphurea*	0	0	0	0	0	0	0	0	0	0	0	0	0	3	0	0	0	0	0	0	0	0	0	0
		Heptageniidae Gen. sp.	0	0	0	0	0	0	0	0	0	0	0	0	0	0	0	0	0	0	0	0	0	0	5	0
		Rhithrogena semicolorata-Gr.	0	0	0	0	0	0	0	0	0	0	0	0	0	0	0	0	0	0	0	0	0	0	0	0
	Leptophlebiidae	*Paraleptophlebia submarginata*	0	0	0	0	0	0	0	0	0	0	0	0	0	0	0	0	0	0	0	0	0	0	0	0
	Palingeniidae	*Palingenia longicauda*	0	0	0	0	0	0	0	0	7	0	0	0	0	0	0	0	0	0	0	0	0	0	0	0
	Potamantidae	*Potamanthus luteus*	0	0	0	0	0	0	0	0	2	0	0	0	0	0	0	0	0	0	0	0	0	0	0	0
Heteroptera	Aphelocheiridae	*Aphelocheirus aestivalis*	0	0	0	12	0	98	45	2	0	0	0	0	0	3	72	430	15	0	65	0	93	30	0	2
	Corixidae	*Corixidae Gen. sp.*	0	0	0	0	0	0	0	0	0	0	0	54	0	0	0	0	0	0	0	0	0	0	0	0
Odonata	Calopterygidae	*Calopteryx splendens*	0	3	0	36	0	4	10	8	0	10	0	6	0	0	0	0	4	0	0	0	33	17	5	5
	Coenagrionidae	*Coenagrion hylas ssp.*	0	0	0	0	0	0	0	0	0	0	0	0	0	0	0	0	0	0	0	0	0	0	0	5
		Coenagrion mercuriale	0	0	0	0	0	0	0	0	0	0	0	0	4	0	0	0	0	0	0	0	0	0	0	0
		Coenagrion puella/pulchellum	0	0	0	0	0	0	0	0	0	0	0	0	0	0	0	0	0	0	0	0	0	0	0	0
		Coenagrion sp.	0	0	0	0	0	0	0	0	0	0	0	0	0	0	6	0	0	0	0	0	0	4	5	0
	Cordulegastridae	*Cordulegaster sp.*	0	0	0	0	0	0	0	0	2	0	0	0	0	0	0	0	0	0	0	0	0	0	0	0
	Corduliidae/Libellu-idae	*Corduliidae/Libellulidae Gen. sp.*	0	0	0	0	0	0	0	0	0	0	0	12	0	0	0	0	0	0	0	0	0	0	0	0
	Gomphidae	*Gomphidae Gen. sp.*	0	0	0	0	0	0	0	0	1	0	0	0	0	0	6	0	0	0	0	0	0	0	0	0
		Gomphus pulchellus	0	0	0	0	0	0	0	0	0	0	0	0	0	0	6	0	0	0	0	0	0	0	0	0
		Gomphus sp.	0	0	0	0	5	0	0	0	0	0	0	0	0	0	0	0	0	0	0	0	0	0	0	0

		Gomphus vulgatissimus	0	0	0	0	0	1	0	0	0	0	0	0	8	0	0	0	0	0	0	0	0	0	0	0
		Onychogomphus forcipatus forcipatus	0	0	0	0	0	2	0	0	0	0	0	0	0	0	6	5	4	6	0	0	0	0	0	0
		Ophiogomphus cecilia	0	0	0	0	0	0	0	0	0	0	0	0	0	0	6	0	0	0	0	0	0	0	0	0
	indet.	*Odonata Gen. sp.*	0	0	0	60	0	6	15	2	0	0	1	0	4	0	132	15	0	6	10	3	23	64	260	0
	Libellulidae	*Orthetrum sp.*	0	0	0	0	0	0	0	0	0	0	0	0	0	0	0	0	0	0	0	0	0	0	0	0
	Platycnemididae	*Platycnemis pennipes*	0	0	0	0	5	0	5	2	0	0	0	72	4	0	0	0	0	0	0	0	3	0	5	0
Plecoptera	Chloroperlidae	*Chloroperlidae Gen. sp.*	0	0	0	0	0	0	0	0	0	0	0	0	0	0	0	0	0	0	0	0	0	0	0	0
		Siphonoperla sp.	0	0	0	0	0	0	0	0	0	0	0	0	0	0	0	0	0	0	0	0	0	0	0	0
	Leuctridae	*Leuctra sp.*	0	0	0	0	0	0	0	0	0	0	0	0	0	3	0	0	0	0	0	0	0	0	0	0
	Nemouridae	*Amphinemura sp.*	0	0	0	0	0	0	0	0	0	0	0	0	0	0	0	0	0	0	0	0	0	0	0	0
	Perlodidae	*Isoperla sp.*	0	0	0	0	0	0	0	0	0	0	0	0	0	0	0	0	0	0	0	0	0	0	0	0
	Taeniopterygidae	*Brachyptera risi*	0	0	0	0	0	0	0	0	0	0	0	0	0	0	0	0	0	0	0	0	0	0	0	0
Trichoptera	Hydropsychidae	*Hydropsyche angustipennis angustipennis*	2	0	0	30	0	35	0	0	0	25	0	0	0	0	0	25	15	0	0	3	13	0	15	3
		Hydropsyche bulbifera	0	0	0	0	0	0	0	0	0	0	0	0	0	0	0	0	0	0	0	0	0	0	0	0
		Hydropsyche guttata	0	0	0	3	0	1	0	0	0	0	0	0	0	0	0	0	4	0	0	0	0	0	0	0
		Hydropsyche pellucidula-Gr.	0	0	0	0	0	6	3	0	0	0	0	0	0	10	12	0	4	0	0	0	0	0	0	0
		Hydropsyche saxonica	0	0	0	0	0	0	0	0	0	0	0	0	0	0	0	0	0	0	0	0	0	0	0	0
		Hydropsyche siltalai	0	0	0	0	0	0	0	0	0	0	0	0	0	0	0	0	0	0	0	0	0	0	0	0
		Hydropsyche sp.	0	0	0	3	0	5	0	0	0	0	0	0	0	13	0	5	0	0	0	0	0	9	5	2
	Hydroptilidae	*Hydroptila sp.*	0	0	0	0	0	0	0	0	0	0	0	30	0	0	0	20	4	6	0	9	0	0	60	17
		Hydroptilidae Gen. sp.	0	0	0	0	0	0	0	0	0	0	0	0	0	0	0	0	0	0	0	3	3	4	0	0
		Stactobia sp.	0	0	0	0	0	0	0	0	0	0	0	0	0	0	0	0	0	0	0	0	0	0	0	0
	Leptoceridae	*Athripsodes albifrons*	0	0	0	0	0	0	3	0	0	0	0	6	8	0	0	0	0	0	0	3	7	0	5	0
		Athripsodes bilineatus bilineatus	0	0	0	0	0	0	0	0	0	0	0	0	0	0	0	0	0	0	0	0	0	0	0	0
		Athripsodes cinereus	0	0	0	0	0	0	0	0	0	0	0	0	0	0	0	0	0	0	0	6	3	0	0	0
		Leptoceridae Gen. sp.	0	1	0	0	0	0	0	12	0	0	0	0	0	0	0	0	0	0	0	3	3	0	0	0
		Mystacides azurea	0	0	0	0	0	1	3	12	0	0	0	54	0	0	0	0	0	0	0	0	0	4	0	3
		Oecetis ochracea	0	0	0	0	0	0	0	0	0	0	0	0	0	0	0	0	0	0	0	0	0	0	0	0
	Lymnephilidae	*Halesus radiatus*	0	0	0	0	0	0	0	0	0	0	0	0	0	0	0	0	0	0	0	0	0	0	0	0
		Limnephilidae Gen. sp.	0	0	0	0	0	0	0	0	0	0	0	0	0	0	0	0	0	0	0	0	0	0	0	0
		Limnephilus borealis	0	0	0	0	0	0	0	0	0	0	0	0	0	0	0	0	0	0	0	0	0	0	0	0
		Limnephilus flavicornis	0	0	0	0	0	0	0	0	0	0	0	0	0	0	0	0	0	0	0	0	0	0	0	0

	Limnephilus lunatus	0	0	0	0	0	0	0	0	3	0	0	0	0	0	0	0	0	0	0	0	0	0	0	0
	Micropterna lateralis	0	0	0	0	0	0	0	0	1	0	0	0	0	0	0	0	0	0	0	0	0	0	0	0
	Pseudopsilopteryx zimmeri	0	0	0	0	0	0	0	0	0	0	0	0	0	0	0	0	0	0	0	0	0	0	0	0
Rhyacophilidae	*Rhyacophila glareosa*	0	0	0	0	0	0	0	0	0	0	0	0	0	0	12	0	0	0	0	0	0	0	0	0
	Rhyacophila (Rhyacophila) sp.	0	0	1	0	0	0	0	0	0	0	0	0	0	33	6	0	0	0	0	0	0	0	0	0
Psychomyiidae	*Lype phaeopa*	0	0	0	0	0	0	0	0	0	0	0	0	0	0	0	0	0	0	0	0	0	0	0	0
	Lype reducta	0	0	0	0	0	0	0	0	0	0	0	0	0	0	0	0	0	0	0	0	0	0	0	0
	Psychomyia pusilla	0	0	0	0	0	3	0	0	0	0	0	0	0	0	0	0	0	0	0	0	0	0	0	0
	Tinodes sp.	0	0	0	0	0	0	0	0	0	0	0	0	0	0	6	0	0	0	0	0	0	0	0	0
