# Supplementary material for: Do Contaminants Originating from State-of-the-Art Treated Wastewater Impact the Ecological Quality of Surface Waters?
Source: PLoS One. 2013 Apr 8;8(4):e60616. doi: 10.1371/journal.pone.0060616 (PMC3620539; doi:10.1371/journal.pone.0060616)
Supplement: Table S3 — List of taxa of aquatic invertebrates found during the sampling campaign in March 2006 at 26 sampling points. (PDF) [file pone.0060616.s008.pdf]

Table S3. List of taxa of aquatic invertebrates found during the sampling campaign in March 2006 at 26 sampling points.

Class	Order	Family	Taxon	La 1	La 2	Mo 1	Mo 2	Mo 3	Mo 4	Sa 1	Sa 2	Sa 3	Sw 1	Sw 2	Sw 3	Sw 4	Sw 5	Sw 6	We 1	We 2	We 3	We 4	We 5	We 6	Wi 1	Wi 2	Wi 3	Wi 4	Wi 5
Cestoda		indet.	*Cestoda Gen. sp.*	0	0	0	0	0	0	0	0	0	0	0	0	0	0	0	0	0	0	0	0	0	0	0	0	0	3
Nematoda		indet.	*Nematoda Gen. sp.*	0	0	0	0	0	0	0	0	6	0	0	4	0	6	0	0	0	0	0	0	0	54	6	15	18	11
Turbellaria	Tricladida	Dendrocoelidae	*Dendrocoelum lacteum*	0	0	42	0	0	0	0	0	0	0	0	0	0	0	0	0	0	0	0	0	0	0	0	0	0	0
		Dugesiidae	*Dugesia sp.*	10	0	0	0	0	0	0	0	0	0	12	13	18	0	0	0	0	0	0	0	0	0	0	0	0	0
		Planariidae	*Planaria sp.*	0	0	0	0	0	0	0	0	0	0	0	9	0	30	7	0	0	0	0	12	0	0	0	4	0	0
Bivalvia	Eulamellibranchiata	Corbiculidae	*Corbicula fluminea*	0	20	0	0	0	43	0	0	0	0	0	56	18	66	13	0	0	0	0	0	36	0	0	0	48	104
		Sphaeriidae	*Musculium transversum*	0	10	0	0	0	0	0	0	0	0	0	4	18	66	0	0	0	0	0	0	0	0	0	0	0	0
			Pisidium sp.	0	15	18	33	70	9	6	0	0	12	24	81	24	108	3	0	8	4	6	48	0	72	60	26	36	5
			Sphaerium corneum	0	0	0	0	0	0	0	0	0	0	0	0	0	0	0	0	0	0	0	0	0	0	0	0	0	0
			Sphaerium rivicola	0	0	0	0	0	0	0	0	0	0	0	0	0	0	0	0	0	0	0	0	0	0	0	0	0	0
			Sphaerium sp.	0	0	42	0	10	0	18	0	6	0	0	0	12	0	0	9	14	266	54	72	0	0	0	0	0	0
		Unionidae	*Unio pictorum ssp.*	0	0	0	0	5	0	0	0	0	0	0	0	0	0	0	0	0	0	0	0	0	0	0	0	0	0
Gastropoda	Prosobranchia	Bithyniidae	*Bithynia tentaculata*	0	0	0	0	0	0	0	0	0	0	0	0	6	12	0	0	0	11	0	0	0	0	0	0	0	0
		Hydrobiidae	*Potamopyrgus antipodarum*	0	65	0	0	90	21	0	1140	6	18	366	13	6	258	33	0	23	11	0	0	0	90	0	0	24	22
	Basommatophora	Ancylidae	*Ancylus fluviatilis*	0	0	36	54	20	21	90	18	18	6	0	0	0	0	3	7	17	49	0	0	0	18	18	0	0	11
		Lymnaeidae	*Galba truncatula*	0	0	0	3	0	0	0	0	0	12	0	0	0	0	0	0	2	0	0	0	0	0	18	4	0	0
			Lymnaeidae Gen. sp.	0	0	0	0	0	0	0	0	0	0	0	0	0	0	0	0	0	0	0	0	0	0	0	0	0	0
			Radix auricularia	0	0	0	0	0	0	0	0	0	0	0	0	0	0	0	0	0	0	0	0	0	0	0	0	0	0
			Radix balthica	0	0	0	0	0	0	0	0	0	0	0	0	0	0	0	0	0	4	0	0	0	0	36	0	0	0
			Radix sp.	0	0	0	0	0	0	0	0	0	0	0	0	0	6	0	0	2	0	0	0	0	0	0	26	6	5
			Stagnicola sp.	0	0	0	0	0	0	0	0	0	0	0	0	0	0	0	0	0	0	0	0	0	0	0	0	0	3
		Physidae	*Physa fontinalis*	0	0	0	0	0	0	0	0	6	0	0	0	0	0	0	0	0	0	0	0	0	0	0	0	0	0

			Physella sp.	235	30	0	0	0	4	0	0	0	0	0	9	0	0	7	0	0	0	6	0	0	0	0	15	0	0
			Physidae Gen. sp.	0	0	0	0	0	0	0	0	0	0	0	0	0	6	0	0	0	0	0	0	0	0	0	0	0	0
		Planorbidae	*Bathyomphalus contortus*	0	0	0	0	0	0	0	0	0	0	0	0	6	0	0	0	0	0	0	0	0	0	0	0	0	0
			Gyraulus sp.	0	0	0	0	0	0	0	0	0	0	0	0	0	0	0	0	0	0	0	0	0	0	0	0	0	0
			Planorbidae Gen. sp.	0	0	0	0	0	0	0	0	0	0	0	0	0	0	0	0	0	0	0	0	0	0	0	0	0	0
Annelida	Oligochaeta	indet.	*Oligochaeta Gen. sp.*	4999	90	102	639	330	596	90	510	222	90	180	274	144	300	297	180	204	244	498	330	162	654	960	244	318	109
	Hirudinea	Erpobdellidae	*Erpobdella octoculata*	0	0	72	12	5	0	6	0	0	0	0	4	12	18	0	2	5	8	0	12	24	48	18	0	0	0
		Glossiphoniidae	*Glossiphonia complanata*	0	0	12	0	0	0	0	0	0	0	0	0	0	0	0	0	0	0	0	0	0	6	6	0	0	0
			Glossiphonia concolor	0	0	0	0	0	0	6	0	0	0	0	0	0	0	0	0	0	0	0	0	0	6	0	0	0	0
			Glossiphonia verrucata	0	0	0	0	0	0	0	0	0	0	0	0	0	0	0	0	0	0	0	0	0	0	0	0	0	0
			Helobdella stagnalis	80	0	0	9	5	0	6	0	0	0	0	0	12	0	0	0	0	8	6	0	0	0	0	0	42	5
		Piscicolidae	*Piscicolidae Gen. sp.*	0	0	0	0	0	0	0	0	0	0	0	0	0	0	0	0	0	0	0	0	0	0	0	0	0	0
Crustacea	Amphipoda	Crangonycitidae	*Crangonyx pseudogracilis*	0	0	0	0	0	0	0	0	0	0	0	0	0	0	0	0	0	0	0	0	6	0	0	0	0	0
		Gammaridae	*Dikerogammarus sp.*	0	0	0	0	0	0	0	0	0	0	0	0	0	0	0	0	0	0	0	0	0	0	0	0	0	0
			Dikerogammarus villosus	0	0	0	0	0	0	0	0	0	0	0	0	0	0	0	0	0	0	0	0	0	0	0	0	0	35
			Gammarus fossarum	0	0	0	0	0	0	0	0	0	0	0	0	0	0	0	2	0	0	0	0	0	0	0	0	0	0
			Gammarus lacustris	0	0	0	6	0	0	0	0	0	0	0	0	0	0	0	0	0	0	0	0	0	0	0	0	0	0
			Gammarus pulex	0	0	1548	18	20	9	30	24	18	60	0	0	0	0	0	0	0	0	0	0	0	18	0	0	0	0
			Gammarus roeselii	0	30	0	90	0	0	12	6	0	234	924	0	636	654	0	2	9	11	12	6	24	0	0	0	0	0
			Gammarus sp.	0	0	390	57	65	21	66	24	42	48	0	30	0	0	17	0	0	0	0	0	0	0	0	0	0	0
			Gammarus tigrinus	0	70	0	0	0	0	0	0	0	0	0	206	30	0	463	0	0	0	0	0	0	0	0	0	0	0
	Isopoda	Asellidae	*Asellus aquaticus*	45	210	0	0	15	4	0	18	126	54	18	56	78	54	87	0	0	19	12	90	138	6	18	0	234	63
Insecta	Coleoptera	Dytiscidae	*Platambus maculatus Lv.*	0	0	0	0	0	0	0	0	0	0	0	0	0	0	0	0	0	0	0	0	0	18	0	0	0	0
		Elmidae	*Elmis aenea/maugetii/rietscheli/rioloides Ad.*	0	0	0	0	0	0	0	12	6	6	0	0	12	0	0	1	0	0	0	0	0	0	0	0	6	0

		Elmis latreillei Lv.	0	0	0	0	0	0	12	0	0	0	0	0	0	0	0	0	0	0	0	0	0	0	0	0	0	0
		Elmis maugetii Lv.	0	0	0	0	0	0	24	0	0	0	6	0	0	0	0	0	4	0	0	0	12	42	12	8	12	0
		Elmis rietscheli Lv.	0	0	0	9	5	13	36	0	12	12	42	0	0	0	0	9	2	11	18	6	30	18	0	0	18	0
		Elmis rioloides Lv.	0	0	0	0	0	0	0	0	0	0	0	0	0	0	0	0	0	0	0	0	0	12	0	0	0	0
		Elmis sp. Lv.	0	0	0	0	0	0	0	0	0	0	0	0	0	0	0	0	0	0	0	0	0	0	0	0	0	0
		Limnius muelleri	0	0	0	0	0	0	0	0	0	0	0	0	0	0	0	0	0	0	0	0	0	0	0	0	0	0
		Limnius volckmari Ad.	0	0	0	0	0	0	0	0	0	0	0	0	0	0	0	0	0	4	0	0	0	0	0	0	0	0
		Limnius volckmari Lv.	0	0	0	0	0	0	0	0	0	0	0	0	0	0	0	33	4	15	18	66	6	72	6	0	0	0
		Oulimnius sp. Ad.	0	0	0	0	0	0	0	0	0	0	0	0	0	0	0	0	0	0	0	6	18	0	0	0	0	3
		Oulimnius tuberculatus Lv.	0	0	0	0	0	0	0	0	0	0	0	13	0	0	0	0	1	0	30	24	18	0	18	0	6	0
	Georissidae	*Georissus sp. Ad.*	0	0	0	0	0	0	0	0	0	0	0	0	0	0	0	0	0	0	0	0	0	0	0	0	0	0
	Gyrinidae	*Gyrinidae Gen. sp. Lv.*	0	0	0	0	0	0	0	0	0	0	0	0	0	0	0	0	0	0	0	0	0	0	0	0	0	0
		Orectochilus villosus Lv.	0	0	0	0	0	0	6	0	0	0	0	0	0	0	0	0	0	0	0	0	0	0	0	0	0	0
	Haliplidae	*Haliplus (Haliplus) sp. Ad.*	0	0	0	0	0	0	0	0	0	0	0	0	0	0	0	0	0	4	0	0	0	0	0	0	0	0
		Haliplus (Liaphlus) sp. Ad.	0	0	0	0	0	0	0	0	0	0	0	0	0	0	0	0	0	0	0	0	0	0	0	0	0	0
		Haliplus lineatocollis Lv.	0	0	0	0	0	0	0	0	0	0	0	0	0	0	0	0	0	0	0	0	0	0	0	0	0	0
		Haliplus sp. Lv.	0	0	0	0	0	0	0	0	0	0	0	0	6	0	0	0	0	0	0	0	0	0	18	4	24	0
	Hydrophilidae	*Laccobius sp. Lv.*	0	0	0	3	0	0	0	0	6	0	6	0	6	0	0	0	0	0	0	0	0	0	0	0	0	0
	indet.	*Coleoptera Gen. sp. Lv.*	0	0	0	0	0	0	0	0	0	12	0	0	0	0	0	0	0	0	0	0	0	0	0	0	0	0
	Psephenidae	*Eubria palustris Lv.*	0	0	0	0	0	0	0	0	0	0	0	0	0	0	0	0	0	0	0	6	0	0	0	0	0	0
Diptera	Ceratopogonidae	*Ceratopogoninae/Palpomyiinae Gen. sp.*	0	0	0	30	145	26	42	60	54	0	6	13	12	12	3	15	14	30	132	42	96	312	312	26	138	46
	Chironomidae	*Chironominae Gen. sp.*	15	0	6	18	15	47	18	30	126	48	0	4	24	0	0	3	8	360	780	678	361	0	546	169	18	76
		Chironomini Gen. sp.	15	0	0	9	35	43	18	36	126	240	18	26	6	42	27	3	16	38	120	109	66	0	192	23	48	0
		Tanytarsini Gen. sp.	0	30	0	0	260	39	138	204	162	60	24	120	42	6	7	7	14	34	570	942	726	294	258	308	870	177

		Prodiamesa olivacea	0	575	18	174	275	197	0	186	1044	6	84	227	318	78	87	14	51	34	246	42	12	1104	948	660	24	22
		Tanypodinae Gen. sp.	0	35	0	6	0	13	30	348	126	42	54	64	30	6	3	0	3	19	30	210	318	120	48	23	6	3
		Orthocladiinae Gen. sp.	170	65	18	234	435	9	378	1080	216	228	78	64	6	18	0	17	39	270	336	1146	516	504	378	94	396	368
	Culicidae	*Culicidae Gen. sp.*	0	0	0	0	0	0	0	0	0	0	0	0	0	0	0	0	0	0	0	0	0	0	0	0	0	0
	Dolichopodidae	*Dolichopodidae Gen. sp.*	0	0	0	0	0	0	0	0	0	6	0	0	12	0	3	0	1	0	0	0	0	0	0	0	0	0
	Empididae	*Chelifera sp.*	0	0	0	3	0	0	0	6	0	0	0	0	0	0	0	0	0	0	0	24	30	12	6	0	6	0
		Clinocerinae Gen. sp.	0	0	0	3	0	0	6	0	0	0	0	0	0	0	0	0	0	0	0	0	0	6	0	0	0	0
		Hemerodromia sp.	0	0	0	0	0	0	0	6	0	0	0	0	0	0	0	3	0	0	0	6	0	18	6	0	0	0
	indet.	*Diptera Gen. sp.*	0	0	0	0	0	0	0	0	0	0	0	0	0	0	0	0	0	0	0	0	0	0	0	0	0	0
	Limoniidae/Pediciidae	*Dicranota sp.*	0	0	18	0	0	0	0	12	0	0	6	0	0	0	0	5	6	0	0	6	0	6	60	0	0	0
		Limoniidae Gen. sp.	0	0	0	3	0	0	0	0	0	6	0	0	12	0	0	0	0	4	0	0	0	6	0	0	6	0
	Muscidae	*Lispe sp.*	0	0	0	3	0	0	0	0	0	0	0	0	0	0	0	0	1	0	0	0	0	24	0	4	0	0
	Psychodidae	*Psychodidae Gen. sp.*	0	0	6	0	0	0	0	0	0	6	0	0	0	0	0	0	0	0	0	0	0	72	0	0	0	3
	Ptychopteridae	*Ptychoptera sp.*	0	0	0	0	0	0	0	0	0	0	0	0	0	0	0	0	0	0	0	0	6	0	0	0	0	0
	Rhagionidae	*Chrysopilus sp.*	0	0	0	0	0	0	0	0	0	0	0	0	0	0	0	0	0	0	0	0	0	0	0	0	0	0
	Sciomyzidae	*Sciomyzidae Gen. sp.*	0	0	0	0	0	0	0	0	0	0	0	0	0	0	3	0	0	0	0	0	0	0	0	0	0	0
	Simuliidae	*Simulium sp.*	5	15	0	0	0	4	12	18	24	0	18	0	0	6	0	2	8	75	36	342	96	66	54	0	6	8
	Stratiomyiidae	*Stratiomyiidae Gen. sp.*	0	0	0	0	0	0	0	0	24	0	0	0	0	0	0	0	2	8	6	0	0	0	0	0	0	0
	Tabanidae	*Chrysops sp.*	0	0	0	0	0	0	0	0	0	6	6	0	0	6	0	0	0	0	0	0	6	0	42	4	6	0
		Tabanidae Gen. sp.	0	0	0	0	10	0	0	0	0	0	0	0	0	0	0	1	0	45	0	0	0	6	0	0	0	0
	Tipulidae	*Tipula sp.*	0	0	12	9	0	0	24	0	0	12	0	0	0	0	0	0	0	4	0	0	0	0	0	0	0	0
Ephemeroptera	Baetidae	*Baetis rhodani*	0	0	0	0	0	0	36	0	0	0	0	0	0	0	0	0	0	0	0	0	0	0	0	0	0	0
		Baetis sp.	0	0	348	45	50	0	66	30	18	0	0	0	0	0	0	37	2	4	6	12	0	60	42	0	24	11
		Cloeon dipterum	0	0	0	0	0	0	0	0	0	0	0	0	0	0	33	0	0	0	0	0	0	0	0	0	0	0

	Caneidae	*Caenis pusilla*	0	0	0	0	0	0	0	0	0	0	0	0	0	0	0	0	0	0	0	0	0	6	0	0	0	0
		Caenis sp.	0	0	0	0	5	9	6	60	18	0	0	4	0	0	0	0	1	0	6	0	0	0	282	4	96	8
	Ephemerellidae	*Ephemerella sp.*	0	0	0	0	0	0	0	0	0	0	0	0	0	0	0	0	0	0	0	0	0	0	0	0	0	0
	Ephemeridae	*Ephemera danica*	0	0	0	0	0	0	0	0	0	156	0	0	0	0	0	1	2	0	0	0	0	6	324	23	66	16
	Heptageniidae	*Heptagenia sulphurea*	0	0	0	0	0	0	0	0	0	0	0	0	0	0	0	1	0	0	0	0	0	0	0	0	0	0
		Heptageniidae Gen. sp.	0	0	0	0	0	0	0	6	0	0	0	0	0	0	0	0	0	0	0	0	0	0	0	0	0	0
		Rhithrogena semicolorata-Gr.	0	0	0	0	0	0	0	0	0	0	0	0	0	0	0	9	0	0	0	0	0	0	0	0	0	0
	Leptophlebiidae	*Paraleptophlebia submarginata*	0	0	0	0	0	0	0	0	0	54	0	0	0	0	0	0	0	0	0	0	0	0	0	0	0	0
	Palingeniidae	*Palingenia longicauda*	0	0	0	0	0	0	0	0	0	0	0	0	0	0	0	0	0	0	0	0	0	0	0	0	0	0
	Potamantidae	*Potamanthus luteus*	0	0	0	0	0	0	0	0	0	0	0	0	0	0	0	0	0	0	0	0	0	0	0	0	0	0
Heteroptera	Aphelocheiridae	*Aphelocheirus aestivalis*	0	0	0	18	5	9	138	48	0	0	0	0	0	0	0	0	7	19	108	24	42	0	168	34	0	0
	Corixidae	*Corixidae Gen. sp.*	0	0	0	0	0	0	0	0	0	0	0	0	6	0	0	0	0	0	0	0	0	0	0	4	0	0
Odonata	Calopterygidae	*Calopteryx splendens*	0	0	0	0	0	0	0	12	12	0	6	13	18	0	0	0	0	0	0	18	24	0	0	0	30	0
	Coenagrionidae	*Coenagrion hylas ssp.*	0	0	0	0	0	0	0	0	0	0	0	0	0	0	0	0	0	0	0	0	0	0	0	0	0	0
		Coenagrion mercuriale	0	0	0	0	0	0	0	0	0	0	0	0	0	0	0	0	0	0	0	0	0	0	0	0	0	0
		Coenagrion puella/pulchellum	0	0	0	0	0	0	0	0	0	6	0	0	0	0	0	0	0	0	0	0	0	0	0	4	0	0
		Coenagrion sp.	0	10	0	0	0	0	0	0	0	0	0	0	0	0	0	0	0	0	0	0	0	0	0	0	0	0
	Cordulegastridae	*Cordulegaster sp.*	0	0	0	0	0	0	0	0	0	0	0	0	0	0	0	0	0	0	0	0	0	0	0	0	0	0
	Corduliidae/Libellulidae	*Corduliidae/Libellulidae Gen. sp.*	0	0	0	0	0	0	0	0	0	0	0	0	0	0	0	0	0	0	0	0	0	0	0	0	0	0
	Gomphidae	*Gomphidae Gen. sp.*	0	0	0	0	0	0	0	0	0	0	0	0	0	0	0	0	0	0	0	0	0	0	0	0	0	0
		Gomphus pulchellus	0	0	0	0	0	0	0	0	0	0	0	0	0	0	0	0	0	0	0	0	0	0	0	0	0	0
		Gomphus sp.	0	0	0	0	0	0	0	0	0	0	0	0	0	0	0	0	0	0	0	0	0	0	0	0	0	0
		Gomphus vulgatissimus	0	0	0	0	0	0	0	0	0	0	6	0	6	0	0	0	0	0	0	0	0	0	0	0	0	3
		Onychogomphus forcipatus forcipatus	0	0	0	0	0	0	6	0	0	0	6	0	0	0	0	0	7	0	12	24	0	0	0	0	0	0

		Ophiogomphus cecilia	0	0	0	0	0	0	0	0	0	0	0	0	0	0	0	0	1	4	0	0	0	0	0	0	0	0
	indet.	*Odonata Gen. sp.*	0	0	0	0	0	0	0	0	0	0	0	0	0	0	0	0	0	0	0	0	0	0	0	0	0	0
	Libellulidae	*Orthetrum sp.*	0	0	0	0	0	0	0	0	0	0	0	0	12	0	0	0	0	0	0	0	6	0	0	4	0	0
	Platycnemididae	*Platycnemis pennipes*	0	0	0	0	0	0	0	0	0	0	0	0	0	0	0	0	0	0	0	0	0	0	0	0	0	0
Plecoptera	Chloroperlidae	*Chloroperlidae Gen. sp.*	0	0	0	0	0	0	0	0	0	0	0	0	0	0	0	1	0	0	0	0	0	0	0	0	0	0
		Siphonoperla sp.	0	0	0	0	0	0	0	0	0	6	0	0	0	0	0	0	0	0	0	0	0	0	0	0	0	0
	Leuctridae	*Leuctra sp.*	0	0	0	0	0	0	0	0	0	0	0	0	0	0	0	0	0	0	0	0	0	0	0	0	0	0
	Nemouridae	*Amphinemura sp.*	0	0	0	0	0	0	0	0	0	12	0	0	0	0	0	0	0	0	0	0	0	0	0	0	0	0
	Perlodidae	*Isoperla sp.*	0	0	0	0	0	0	0	0	0	30	0	0	0	0	0	0	0	0	0	0	0	0	0	0	0	0
	Taeniopterygidae	*Brachyptera risi*	0	0	0	0	0	0	0	0	0	102	0	0	0	0	0	1	0	0	0	0	0	0	0	0	0	0
Trichoptera	Hydropsychidae	*Hydropsyche angustipennis angustipennis*	205	0	0	6	10	0	600	48	48	6	12	0	6	6	0	0	1	41	30	18	6	18	54	0	96	11
		Hydropsyche bulbifera	0	0	0	0	0	0	0	0	0	0	0	0	0	0	0	0	2	0	6	0	0	0	0	0	0	0
		Hydropsyche guttata	0	0	0	0	0	0	0	0	0	0	0	0	0	0	0	0	0	0	0	0	0	0	0	0	0	0
		Hydropsyche pellucidula-Gr.	0	0	0	0	0	0	0	0	0	0	0	0	0	0	0	1	0	0	0	0	0	0	0	0	6	0
		Hydropsyche saxonica	0	0	6	0	0	0	0	0	0	0	0	0	0	0	0	1	1	0	0	0	0	0	0	0	0	0
		Hydropsyche siltalai	0	0	6	0	0	0	0	0	0	54	0	0	0	0	0	6	0	0	0	0	0	0	0	0	0	0
		Hydropsyche sp.	0	0	0	0	0	0	0	0	0	0	0	0	0	0	0	0	0	0	0	0	0	0	0	0	0	0
	Hydroptilidae	*Hydroptila sp.*	0	0	0	0	0	0	0	0	12	0	0	0	0	0	0	0	0	0	0	0	6	6	12	0	18	0
		Hydroptilidae Gen. sp.	0	0	0	0	0	0	0	0	0	0	0	0	0	0	0	0	0	0	0	0	0	0	0	0	0	0
		Stactobia sp.	0	0	0	0	0	0	0	0	0	0	0	0	0	0	0	0	0	0	6	0	0	6	6	0	18	0
	Leptoceridae	*Athripsodes albifrons*	0	0	0	0	0	0	0	0	0	0	0	0	0	0	0	0	0	0	0	6	0	0	0	0	0	0
		Athripsodes bilineatus bilineatus	0	0	0	0	0	0	0	0	0	0	6	4	0	0	0	0	0	0	0	6	0	30	66	0	12	3
		Athripsodes cinereus	0	0	0	0	0	0	0	0	0	0	0	0	0	0	0	0	0	0	0	0	0	0	0	0	0	0
		Leptoceridae Gen. sp.	0	0	0	0	0	0	0	0	0	0	0	0	0	0	0	0	0	0	0	0	0	0	0	0	0	0

	Mystacides azurea	0	0	0	0	0	0	0	0	0	6	0	0	0	0	0	0	0	0	0	0	12	18	6	4	6	0
	Oecetis ochracea	0	0	0	0	0	0	0	0	0	0	0	0	0	0	0	0	0	0	6	0	0	0	0	0	0	0
Lymnephilidae	*Halesus radiatus*	0	0	0	0	0	0	0	0	0	24	0	0	0	0	0	0	0	0	0	0	0	0	0	0	0	0
	Limnephilidae Gen. sp.	0	0	0	0	0	0	0	0	0	78	0	0	0	0	0	0	0	0	0	0	0	0	0	0	0	0
	Limnephilus borealis	0	0	0	0	0	0	0	0	0	0	0	0	0	0	0	0	0	4	0	0	0	0	0	0	0	0
	Limnephilus flavicornis	0	0	0	0	0	0	0	0	0	0	0	0	0	6	0	0	0	0	0	0	0	0	0	0	0	0
	Limnephilus lunatus	0	0	0	0	0	0	0	0	0	0	0	0	0	0	0	0	0	0	0	0	0	0	0	0	6	0
	Micropterna lateralis	0	0	0	0	0	0	0	0	0	24	0	0	0	0	0	0	0	0	0	0	0	0	0	0	0	0
	Pseudopsilopteryx zimmeri	0	0	0	0	0	0	0	0	0	0	0	0	0	0	0	1	0	0	0	0	0	0	0	0	0	0
Rhyacophilidae	*Rhyacophila glareosa*	0	0	30	3	0	0	12	0	6	0	0	0	0	0	0	1	0	0	0	0	0	0	0	0	0	0
	Rhyacophila (Rhyacophila) sp.	0	0	0	0	0	0	0	0	0	0	0	0	0	0	0	0	0	0	0	0	0	0	0	0	0	0
Psychomyiidae	*Lype phaeopa*	0	0	0	0	0	0	0	0	0	0	0	0	0	0	0	0	0	0	0	12	30	0	0	0	0	0
	Lype reducta	0	0	0	0	0	0	0	0	0	12	0	0	0	0	0	0	0	0	0	0	0	0	0	0	0	0
	Psychomyia pusilla	0	0	0	3	0	9	42	12	0	0	0	0	0	0	0	0	0	0	0	0	0	0	0	0	6	8
	Tinodes sp.	0	0	0	0	0	0	0	0	0	0	0	0	0	0	0	0	0	0	0	0	0	0	0	0	0	0
