# Supplementary material for: Do Contaminants Originating from State-of-the-Art Treated Wastewater Impact the Ecological Quality of Surface Waters?
Source: PLoS One. 2013 Apr 8;8(4):e60616. doi: 10.1371/journal.pone.0060616 (PMC3620539; doi:10.1371/journal.pone.0060616)
Supplement: Table S4 — Average concentrations of 12 organic contaminants in the water phase. (PDF) [file pone.0060616.s009.pdf]

Table S4. Average concentrations of 12 organic contaminants in the water phase (ng/L).

	TBP	TCEP	TBEP	TCPP	TDCPP	BPA	NP	OP	HHCB	AHTN	Terbutryn	DEET
La1	499	358	141	1154	230	186	24	79	440	147	65	284
La2	372	393	235	1176	278	22	6	12	211	53	76	372
Mo1	146	31	95	114	41	36	24	23	26	10	34	1
Mo2	232	363	133	752	146	90	48	35	212	57	634	78
Mo3	220	289	108	643	130	13	60	38	153	38	691	189
Mo4	189	263	176	885	116	137	66	30	155	40	491	130
Sa1	232	162	188	479	115	188	74	43	110	29	786	73
Sa2	241	226	197	617	129	13	91	42	117	31	768	77
Sa3	511	278	119	593	101	22	88	36	92	23	568	80
Sw1	94	94	151	327	34	16	77	13	11	4	14	40
Sw2	452	360	341	1039	184	11	38	33	217	56	87	225
Sw3	295	343	289	982	211	10	59	40	173	45	62	236
Sw4	258	306	293	887	179	5	55	28	159	42	68	287
Sw5	264	323	280	912	182	142	52	33	174	49	67	205
Sw6	286	431	215	1203	224	29	54	37	192	55	69	318
We1	116	44	126	215	84	8	19	15	68	18	12	13
We2	107	67	203	182	65	2	54	18	35	10	20	27
We3	148	136	279	733	158	25	44	147	191	45	1153	78
We4	160	121	343	516	93	6	21	52	118	30	590	62
We5	190	123	220	494	88	7	76	87	111	30	594	91
We6	183	124	201	525	97	31	40	56	105	25	600	69
Wi1	110	14	94	46	15	13	66	17	11	7	9	0
Wi2	141	3	51	37	12	0	55	14	5	4	3	2
Wi3	84	29	45	191	25	2	63	13	29	11	11	36
Wi4	138	116	185	500	86	95	35	24	99	25	32	125
Wi5	102	141	141	680	114	146	36	32	105	34	85	207
