# Supplementary material for: Do Contaminants Originating from State-of-the-Art Treated Wastewater Impact the Ecological Quality of Surface Waters?
Source: PLoS One. 2013 Apr 8;8(4):e60616. doi: 10.1371/journal.pone.0060616 (PMC3620539; doi:10.1371/journal.pone.0060616)
Supplement: Table S5 — Average concentrations of 12 heavy metals/metalloids in the sediments. (PDF) [file pone.0060616.s010.pdf]

Table S5. Average concentrations of 12 heavy metals/metalloids in the sediments (mg/kg).

	Al	As	Ba	Cd	Co	Cr	Cu	Fe	Mn	Ni	Pb	Zn
La1	35400	9	412	0.22	10.8	108	46.1	48100	349	29.7	63	349
La2	29300	3.7	331	0.73	6.3	58.3	24.9	7540	209	27	31.1	234
Mo1	52600	3.8	647	2.4	10.1	44.4	19.7	24800	916	18.2	25.7	118
Mo2	43100	3	431	0.12	8.7	50.5	14.8	20500	403	17.4	16.8	113
Mo3	44300	3.15	458	0.1	6.8	53.15	12.05	16800	325.5	16.9	17.25	109
Mo4	45500	4.2	700	0.05	6.1	36.2	14.3	15100	247	13.9	28.8	107
Sa1	63300	3.1	757	0.05	9.4	54.3	13.1	19600	423	22.7	18.2	139
Sa2	51000	5	517	0.1	9.3	44.6	14.3	21000	355	18.9	18	127
Sa3	50000	6.6	517	0.48	12.1	69.5	61.5	22500	460	30.2	76.9	355
Sw1	19200	3.4	417	0.05	1.9	15.8	2.7	2470	19.6	4.6	11.7	19.6
Sw2	17700	2.4	353	0.57	2.8	16.2	13.8	4300	105	5.9	16.7	105
Sw3	25600	3.9	406	0.77	4.5	25.4	18.3	5420	171	16.7	27.1	154
Sw3b	15900	2.3	269	0.33	2.1	10.9	12.2	3280	63.9	5.3	13.1	63.9
Sw4	17800	1.6	441	0.31	1.9	6.9	6.8	2930	58	7.5	13.4	58
Sw5	20100	2.1	271	0.13	2.2	10.2	5.6	2720	43	7.3	14.3	43
We1	60300	10.9	964	0.16	8.6	47.5	17.9	24900	533	15.5	30.5	150
We2	62400	10.7	1240	0.13	8.5	44.8	10.2	24100	636	13.3	80.9	142
We3	67600	10.9	1090	0.03	7.7	40.6	10.4	18400	543	10.6	27.1	117
We4	62100	64.4	1290	0.1	5.8	37.7	7.6	18000	321	8.5	40.3	116
We5	35800	5.8	592	0.19	6.7	48.5	16.9	14100	478	12.9	54.5	133
We6	49000	5.2	1150	0.04	4	18.1	5	9770	343	6.6	28.5	84.2
Wi1	48500	7.4	415	0.49	16	60.1	37.2	24100	514	18.1	39.9	208
Wi2	49300	4.1	393.5	0.22	11.45	36.5	16.05	16050	324	11.3	29.45	117.5
Wi3	56100	4.4	493	0.17	13.1	17.7	8.9	12000	231	10.4	17.9	106
Wi4	45500	6.5	392	0.87	15.3	40.4	19.8	17900	304	15.8	35.4	233
Wi5	29900	3.8	362	0.34	9.5	25.6	16.8	10700	195	39.8	21.5	138
