# Supplementary material for: Do Contaminants Originating from State-of-the-Art Treated Wastewater Impact the Ecological Quality of Surface Waters?
Source: PLoS One. 2013 Apr 8;8(4):e60616. doi: 10.1371/journal.pone.0060616 (PMC3620539; doi:10.1371/journal.pone.0060616)
Supplement: Table S6 — Average concentrations of 16 polycyclic aromatic hydrocarbons in the sediments. (PDF) [file pone.0060616.s011.pdf]

Table S6. Average concentrations of 16 polycyclic aromatic hydrocarbons in the sediments (mg/kg).

	Naph	Acyl	Ace	Fl	Phen	Anth	F	Py	BaA	Chr	BbF	BkF	BaP	DBahA	BghiP	IcdPy
La1	0.04	0	0.04	0.03	1.75	1.01	3.46	2.5	1.53	2.58	1.02	0.66	1.08	0.16	0.4	0.64
La2	0	0	0	0.01	0.04	0.02	0.15	0.11	0.07	0.08	0.07	0.05	0.11	0.01	0.05	0.08
Mo1	0.01	0.01	0.01	0.03	0.08	0.07	0.15	0.11	0.05	0.09	0.05	0.03	0.05	0.01	0.03	0.04
Mo2	0	0	0	0	0.02	0.01	0.05	0.05	0.02	0.06	0.03	0.02	0.03	0	0.02	0.02
Mo3	0.01	0	0.02	0.01	0.79	0.32	2.72	1.98	0.89	1.11	0.53	0.35	0.68	0.07	0.16	0.31
Mo4	0	0	0	0.01	0.01	0	0.07	0.04	0.02	0.01	0.04	0.02	0.03	0.02	0.02	0.02
Sa1	0	0	0	0.01	0.1	0.04	0.2	0.14	0.09	0.1	0.07	0.05	0.07	0.01	0.04	0.06
Sa2	0.01	0.01	0	0.01	0.03	0.01	0.08	0.07	0.04	0.05	0.04	0.03	0.05	0.01	0.03	0.05
Sa3	0.01	0.01	0.02	0.02	0.01	0.08	0.4	0.28	0.12	0.16	0.09	0.09	0.12	0.03	0.05	0.08
Sw1	0	0	0	0	0.01	0.01	0.01	0.01	0.01	0.05	0.01	0	0.01	0	0.01	0.01
Sw2	0.01	0	0.01	0.01	0.06	0.02	0.12	0.09	0.04	0.04	0.04	0.02	0.03	0	0.03	0.05
Sw3	0	0	0	0.01	0.01	0.01	0.04	0.02	0.01	0.02	0.02	0.02	0.03	0	0.02	0.02
Sw3b	0	0	0	0.01	0.01	0.01	0.04	0.02	0.01	0.02	0.02	0.02	0.03	0	0.02	0.02
Sw4	0	0	0	0	0	0	0.01	0.01	0.01	0.01	0.01	0	0.01	0	0	0.01
Sw5	0	0	0	0	0.02	0.01	0.03	0.03	0.01	0.02	0.02	0.01	0.02	0	0.02	0.02
We1	0.01	0.01	0	0.01	0.01	0.02	0.14	0.12	0.07	0.1	0.05	0.05	0.09	0.01	0.04	0.05
We2	0	0.01	0	0.01	0.04	0.02	0.09	0.07	0.04	0.08	0.03	0.03	0.05	0.01	0.02	0.03
We3	0	0	0	0	0.02	0.01	0.04	0.17	0.01	0.04	0.01	0.01	0.02	0	0.01	0.01
We4	0	0	0	0.01	0	0.01	0	0.02	0.01	0.01	0.01	0.02	0.01	0	0.01	0.01
We5	0.01	0.02	0	0.02	0.02	0.01	0.05	0.04	0.02	0.02	0.03	0.03	0.02	0.01	0.02	0.02
We6	0	0	0	0	0.03	0.01	0.15	0.13	0.09	0.11	0.06	0.04	0.09	0.01	0.04	0.05
Wi1	0.01	0.01	0.01	0.01	0.01	0.01	0.01	0.12	0.06	0.03	0.05	0.05	0.06	0.01	0.04	0.05
Wi2	0	0.01	0.01	0.07	0.05	0.05	0.21	0.16	0.09	0.11	0.07	0.06	0.08	0.01	0.04	0.06
Wi3	0	0	0	0.03	0.01	0.01	0.04	0	0.05	0.06	0.04	0.03	0.07	0.01	0.04	0.04
Wi4	0	0.01	0	0.01	0.01	0	0.07	0.05	0.03	0.01	0.03	0.04	0.06	0.01	0.03	0.03
Wi5	0.01	0.01	0	0.01	0.03	0.02	0.08	0.06	0.04	0.07	0.04	0.03	0.06	0.01	0.05	0.03
