# Supplementary material for: Do Contaminants Originating from State-of-the-Art Treated Wastewater Impact the Ecological Quality of Surface Waters?
Source: PLoS One. 2013 Apr 8;8(4):e60616. doi: 10.1371/journal.pone.0060616 (PMC3620539; doi:10.1371/journal.pone.0060616)
Supplement: Table S7 — Loading matrix of principle components calculated by a principle component analysis of organic contaminants listed in Table 2 . (PDF) [file pone.0060616.s012.pdf]

Table S7. Loading matrix of principle components calculated by a principle component analyses of organic contaminants listed in Table 2. Major loading variables on each component are displayed in bold.

	**OC1**	**OC2**	**OC3**	**OC4**
TBP	**0.770**	-0.050	-0.053	0.347
TCEP	**0.906**	-0.094	-0.169	0.296
TBEP	**0.547**	0.198	-0.557	-0.346
TCPP	**0.966**	-0.031	-0.124	0.067
TDCPP	**0.957**	-0.073	-0.152	-0.039
BPA	0.382	0.039	**0.779**	0.098
NP	-0.276	0.400	-0.168	**0.804**
OP	0.357	**0.816**	0.073	-0.307
HHCB	**0.942**	0.088	0.231	-0.070
AHTN	**0.887**	0.020	0.352	-0.052
Terbutryn	0.107	**0.921**	-0.025	0.060
DEET	**0.871**	-0.322	-0.154	0.032
