# Supplementary material for: Do Contaminants Originating from State-of-the-Art Treated Wastewater Impact the Ecological Quality of Surface Waters?
Source: PLoS One. 2013 Apr 8;8(4):e60616. doi: 10.1371/journal.pone.0060616 (PMC3620539; doi:10.1371/journal.pone.0060616)
Supplement: Table S8 — Loading matrix of principle components calculated by a principle component analysis of heavy metals listed in table 2 . (PDF) [file pone.0060616.s013.pdf]

Table S8. Loading matrix of principle components calculated by a principle component analyses of heavy metals listed in table 2. Major loading variables on each component are displayed in bold.

	HM1	HM2	HM3
Al	0.590	**0.688**	0.184
As	0.171	**0.642**	-0.333
Ba	0.218	**0.919**	-0.056
Cd	0.209	-0.277	**0.768**
Co	**0.771**	-0.062	0.245
Cr	**0.898**	-0.125	-0.173
Cu	**0.785**	-0.482	-0.163
Fe	**0.887**	0.129	-0.021
Mn	**0.708**	0.392	0.518
Ni	**0.665**	-0.446	-0.048
Pb	**0.750**	0.121	-0.298
Zn	**0.863**	-0.354	-0.228
