# Supplementary material for: Do Contaminants Originating from State-of-the-Art Treated Wastewater Impact the Ecological Quality of Surface Waters?
Source: PLoS One. 2013 Apr 8;8(4):e60616. doi: 10.1371/journal.pone.0060616 (PMC3620539; doi:10.1371/journal.pone.0060616)
Supplement: Table S9 — Loading matrix of principle components calculated by a principle component analysis of polycyclic aromatic hydrocarbons listed in table 2 . (PDF) [file pone.0060616.s014.pdf]

Table S9. Loading matrix of principle components calculated by a principle component analyses of polycyclic aromatic hydrocarbons listed in table 2. Major loading variables on each component are displayed in bold.

	**PAH1**	**PAH2**
Naph	**0.943**	0.162
Acyl	-0.049	**0.825**
Ace	**0.903**	0.190
Fl	0.293	**0.676**
Phen	**0.991**	-0.065
Anth	**0.985**	-0.003
F	**0.974**	-0.087
Py	**0.972**	-0.089
BaA	**0.995**	-0.061
Chr	**0.994**	-0.053
BbF	**0.998**	-0.045
BkF	**0.998**	-0.014
BaP	**0.991**	-0.057
DBahA	**0.993**	-0.018
BghiP	**0.986**	0.031
IcdPy	**0.997**	-0.026
